# Ring-Augmented Versus Non-Ring Augmented Sleeve Gastrectomy in Patients with BMI > 50 kg/m²: 3-Year Follow-up of a Randomized Controlled Trial

**DOI:** 10.1007/s11695-025-08431-1

**Published:** 2026-01-20

**Authors:** Mohamed Hany, Walid El Ansari, Mohamed H. Zidan, Anwar Ashraf Abouelnasr, Mohamed Ibrahim, Hazem Al Momani, Ala Wafa, Ehab Elmongui, Bart Torensma

**Affiliations:** 1 Department of Surgery, Medical Research Institute, Alexandria University, Alexandria, Egypt; 2 Madina’s Bariatric Center, Madina Women’s Hospital, Alexandria, Egypt; 3 College of Medicine, Ajman University, Ajman, United Arab Emirates; 4 Alexandria University, Alexandria, Egypt; 5 The Research Papyrus Lab, Alexandria, Egypt; 6 Maidstone and Tunbridge Wells NHS Trust, Kent, United Kingdom; 7 NMC Royal Khalifa Hospital, Abu Dhabi, United Arab Emirates; 8 Director of Libyan Board of Metabolic and Bariatric Surgery Fellowship program, Assistant Professor of Surgery, Misurata University, Jamahiriya, Libya; 9 Independent biostatistical consultant, Alexandria, Egypt; 10 Erasmus University Rotterdam, Rotterdam, Netherlands

**Keywords:** Ring-augmented sleeve gastrectomy, Sleeve gastrectomy, Recurrent weight gain, Banded sleeve, Metabolic and bariatric surgery, Food tolerance

## Abstract

**Background:**

Sleeve gastrectomy (SG) is effective but prone to late recurrent weight gain (RWG). Ring-augmented SG (Ra-SG) may preserve restriction and enhance long-term durability.

**Methods:**

In this single-center, prospective, single-blind randomized trial, adults with a BMI > 50 kg/m² were randomized to SG or Ra-SG and followed for 36 months. The primary endpoint was percent total weight loss (%TWL) at 36 months. Secondary outcomes included RWG, gastric pouch volumetry, relapse of associated medical problems, complications, endoscopic findings, laboratory parameters, and patient-reported outcomes (SF-36, Suter).

**Results:**

Ra-SG produced significantly greater weight loss at 36 months (48.8 ± 8.3 vs. 45.5 ± 9.0%TWL; mean difference 3.25% points, 95% CI 0.86 to 5.63; p 0.008). Exploratory analyses showed lower rates of clinically significant RWG (≥ 30% regain: 5.9% vs. 16.3%; absolute risk difference − 10.4%, 95% CI − 19.6 to − 1.2; *p* = 0.033) and smaller pouch volumes (160.1 ± 8.9 vs. 194.2 ± 10.3 mL; *p* < 0.001). Perioperative complications were infrequent and comparable. At three years, the prevalence of endoscopic GERD was similar (17.3% vs. 19.6%). Food tolerance scores were consistently better after SG (all *p* < 0.05). HRQoL improved significantly in both groups, with no differences observed at 36 months. Two Ra-SG patients (1.8%) underwent ring removal.

**Conclusions:**

Ra-SG enhanced weight-loss durability and lowered RWG compared to SG, with similar safety and GERD outcomes but decreased food tolerance at mid-term. Ra-SG may be considered in patients at higher risk of RWG, though multicenter studies with longer follow-up are needed to confirm these findings.

**Supplementary Information:**

The online version contains supplementary material available at 10.1007/s11695-025-08431-1.

## Key Points

1. Ring-augmented Sleeve gastrectomy (Ra-SG) achieved greater %TWL at 3 years vs. Sleeve gastrectomy (+ 3.25% points, 95% CI 0.86–5.63; *p* = 0.008).

2. Recurrent weight gain (RWG) ≥ 30% was less frequent in Ra-SG (5.9% vs. 16.3%; absolute risk difference − 10.4%, 95% CI − 19.6 to − 1.2).

3. Safety and GERD profiles were similar between groups; ring removals were rare (1.8%).

4. Food tolerance was worse after Ra-SG at all follow-ups; counseling on eating behavior and adaptation is essential.

5. Health-Related Quality of Life (HRQoL) improved similarly in both groups; Ra-SG’s main value is weight-loss durability, not incremental HRQoL gains.

**Supplementary Information:**

The online version contains supplementary material available at 10.1007/s11695-025-08431-1.

## Introduction

Obesity, a chronic disease reaching epidemic proportions globally, poses a significant public health challenge and contributes to a myriad of associated diseases, including type 2 diabetes, hypertension, dyslipidemia, and cardiovascular disease [1]. Its escalating prevalence places an immense burden on healthcare systems worldwide, necessitating continuous, effective, and sustainable treatment strategies [1]. While lifestyle modifications and pharmacotherapy play a role, metabolic and bariatric surgery (MBS) remains the most effective long-term treatment for severe obesity, leading to substantial and sustained weight loss, resolution or improvement of obesity-related diseases, and enhanced quality of life [2, 3].

Sleeve gastrectomy (SG) has rapidly emerged as the most performed MBS procedure worldwide, largely due to its technical simplicity, favorable safety profile, and effective weight loss outcomes [4]. SG’s capability to reduce the gastric volume promotes weight loss and alters gut hormone secretion, leading to decreased appetite and increased satiety, which contributes to favorable short- and mid-term outcomes in terms of weight loss and resolution of obesity-related diseases [5, 6]. However, in long-term follow-up studies spanning at least 7 years, the rate of recurrent weight gain (RWG) was found to be 27.8%, with a variability range between 14% and 37%, and an overall revision rate of 19.9%. This includes a specific rate of 13.1% attributed to RWG and 2.9% associated with gastroesophageal reflux disease [7], which can reach 32% at 5 years [8]. This highlights SG’s vulnerability to late RWG, which can reduce long-term metabolic benefits and overall quality of life.

In the realm of severe obesity, defined as BMI > 50 kg/m², the durability of weight-loss outcomes post-SG is less robust than initially indicated. Patients may experience substantial peak weight loss, averaging 30–35% of total body weight (TWL%) at 18 to 24 months, and approximately 22–25% TWL at the five-year mark [9, 10]. Yet, while these figures appear favorable in isolation, they often prove inadequate in sustaining long-term disease remission or preventing relapse of obesity-related conditions in this high-risk cohort.

Comparative studies elucidate this issue: Lemanu et al. highlighted that individuals with severe obesity report greater absolute weight loss, yet exhibit less sustainable relative outcomes compared to those with a BMI below 50 kg/m² [11]. Ece et al. identified a significantly lower percentage of excess weight loss (%EWL) in patients with a BMI of 60 kg/m² or higher [12]. Similarly, Samuel et al. reported a median %EWL of just 39.2% in patients with severe obesity versus 52.6% in those with a BMI between 40 and 49 [13]. Additionally, Khan et al. found that while short-term %TWL was comparable across groups, %EWL remained diminished in those with a BMI > 50 kg/m² [14].

Longitudinal data from Hoyuela further elucidate these concerns, revealing poorer outcomes and elevated revision rates among patients classified with severe obesity [15]. Collectively, SG is particularly susceptible to RWG and relapse of obesity-related conditions in patients with a BMI greater than 50 kg/m², underscoring the need for innovative strategies to enhance the durability of sleeve gastrectomy in this specific subgroup.

Beyond anatomical contributors, several studies have identified patient-level predictors of RWG after SG, such as higher preoperative BMI, older age, obstructive sleep apnea, hepatic steatosis, early insufficient weight loss, emotional eating, and physical inactivity, which heighten the long-term risk of RWG [16–19]. These predictors are especially relevant because they mirror the clinical profile of individuals who may benefit from more durable restrictive reinforcement.

Ring-augmented sleeve gastrectomy (*Ra-*SG), formerly known as banded sleeve gastrectomy [20], has been proposed as a potential enhancement to standard SG by placing a non-adjustable ring around the proximal gastric pouch after sleeve creation, to provide maintained restriction, prevent pouch dilation, and improve long-term weight maintenance; thus decreasing the rate of RWG [21–23].

While preliminary studies and meta-analyses suggest that Ra-SG may lead to superior weight loss outcomes and better weight maintenance compared to standard SG, particularly in the long term, robust evidence from large-scale, randomized controlled trials with extended follow-up periods is still evolving [24, 25]. However, the potential benefits of Ra-SG in terms of weight loss sustainability and resolution of obesity-related diseases need to be carefully weighed against any possible increase in surgical complexity, complications, or adverse events related to the presence of the ring, such as dysphagia or ring erosion [24, 26]. Despite the limited availability of randomized data in patients with severe obesity (BMI ≥ 50 kg/m²), there remains a need to determine whether the anatomical durability offered by Ra-SG translates into superior weight maintenance, sustained control of obesity-related diseases, and an acceptable safety profile in this high-risk group.

This study aims to compare the mid-term efficacy and safety of Ra-SG with the conventional non-ring augmented sleeve gastrectomy (SG) in adults with a BMI > 50 kg/m² over a 36-month follow-up period, focusing on weight loss durability, resolution of obesity-related diseases, complication profile, and patient-reported outcomes, to provide a better understanding of the role of Ra-SG in the management of severe obesity and provide valuable insights for clinical decision-making in MBS.

## Methods

### Trial Design and Registration

This study was conducted as a single-center, prospective, single-blind, parallel-group, randomized controlled trial at the Alexandria Medical Research Institute, Alexandria University, Alexandria, Egypt, a high-volume center for metabolic and bariatric surgery (MBS). The primary objective was to compare the efficacy of ring-augmented sleeve gastrectomy (Ra-SG) against standard sleeve gastrectomy (SG) in adults with a BMI > 50 kg/m² over a follow-up period of 36 months. Participants were administered identical perioperative and postoperative care protocols to ensure consistency between groups.

This trial adhered to an efficacy framework, employing a superiority hypothesis testing strategy. Reporting was conducted in accordance with the CONSORT guidelines [27] and the corresponding extension for randomized trials of non-pharmacologic interventions [28].

The trial was registered at ClinicalTrials.gov with registration number NCT07100327. Ethical approval for the study was granted by the Institutional Review Board of Alexandria Medical Research Institute (Approval Serial Number E/C. S/N. R4/2021; IRB00010526), and the research adhered to the Declaration of Helsinki and its subsequent amendments. Written informed consent was obtained from all participants before their enrollment in the study.

### Participants

Selection criteria for the study included adults aged 18 to 60 years with a body mass index (BMI) of > 50 kg/m² who were candidates for MBS at our high-volume center. Exclusion criteria encompassed a BMI below 50 kg/m², refusal of ring placement, active or previously managed gastroesophageal reflux disease (GERD), presence of a hiatal hernia, history of prior MBS or significant upper gastrointestinal surgeries, plans for pregnancy during the follow-up period, active malignancies, notable psychiatric disorders, substance abuse issues, or any other medical contraindications to surgical intervention.

Baseline preoperative assessments and multidisciplinary evaluation included comprehensive acquisition of medical history, physical examinations, collection of anthropometric data, and documentation of obesity-related diseases, such as type 2 diabetes mellitus, hypertension, dyslipidemia, obstructive sleep apnea, osteoarthritis, and cardiovascular diseases. Laboratory evaluations included hematological, metabolic, nutritional, liver, renal, lipid, and glycemic profiling. All participants underwent standardized Esophagogastroduodenoscopy (EGD) preoperatively to assess esophageal and gastric mucosal integrity, confirm the absence of hiatal hernias, and identify pathological conditions, including severe esophagitis, Barrett’s esophagus, or peptic ulcer disease, conditions that served as further exclusion criteria. No patients were diagnosed with a hiatal hernia preoperatively.

Preoperative quality of life was quantified using the Medical Outcomes Study 36-Item Short-Form Health Survey (SF-36) [29, 30]. Food tolerance was evaluated through the validated Suter questionnaire [31].

**Fig. 1:**
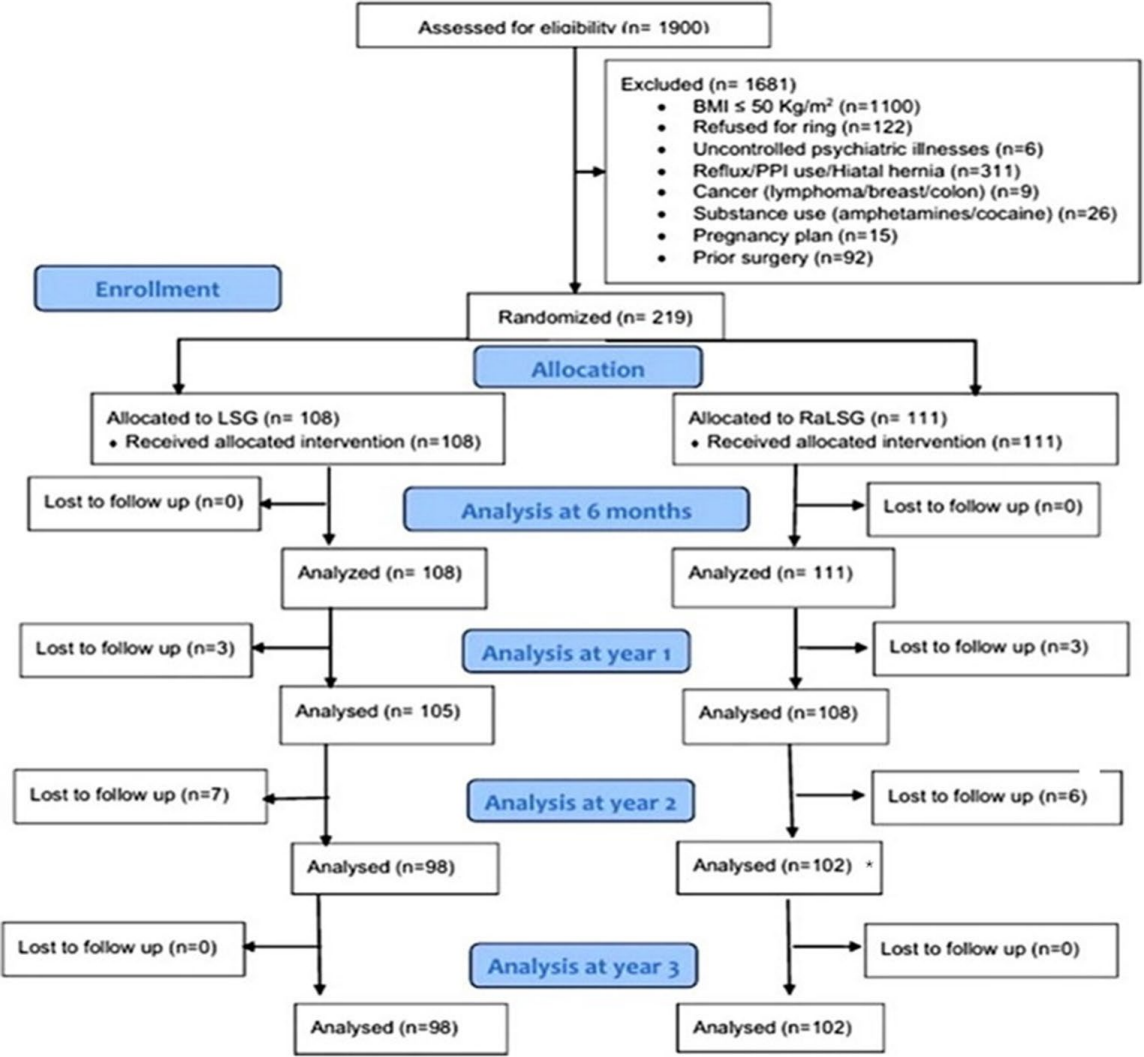
CONSORT flow of participants through the trial. Of 1,900 candidates screened, 1,681 were excluded for predefined reasons. A total of 219 patients were randomized to standard laparoscopic sleeve gastrectomy (SG; *n* = 108) or ring-augmented sleeve gastrectomy (Ra-SG; *n* = 111). Numbers analyzed at each time point were: 6 months: 108 vs. 111; year 1: 105 vs. 108; year 2: 98 vs. 102; year 3: 98 vs. 102, with no additional losses between years 2 and 3. Primary and secondary outcomes were analyzed using the complete data available at each time point. In addition, complete-case analyses across all time points were conducted and are presented in the Supplementary Material. * Two Ra-SG patients underwent ring removal for intolerance but completed follow-up and were analyzed at years 2 and 3 according to their original group assignment (intention-to-treat)

Eligible candidates were consecutively recruited from the outpatient MBS clinic between January 2021 and January 2022; all processes regarding screening, randomization, and follow-up were conducted within a single institution to ensure consistency in eligibility determination, surgical techniques, and perioperative management. The progression of participants through the trial phases, including screening, randomization, intervention allocation, follow-up, and final analysis, is depicted in the CONSORT flow diagram (Fig. 1).

### Randomization, Blinding, and Allocation Concealment

Eligible participants who provided written informed consent were assigned in a 1:1 ratio to undergo either Ra-SG or conventional SG. The random allocation sequence was generated by a statistician not otherwise involved in the trial using a computer-based simple randomization method without stratification. This biostatistician had no role in patient enrollment, clinical management, data collection, or outcome assessment, ensuring independence of sequence generation. Sequentially numbered, opaque, sealed envelopes were prepared by an independent research coordinator, who was not involved in patient recruitment, surgery, or outcome assessment. Participant enrollment was performed by the principal investigator or designated MBS team members. Intervention assignment was carried out in the operating theatre by the attending surgeon only after anesthesia induction, when the sealed envelope corresponding to the participant was opened. This process ensured that allocation concealment was fully maintained until the moment of surgery, preventing any influence on preoperative decision-making or patient counseling.

Given the nature of the surgical intervention, blinding of the operating surgeons and the radiologist interpreting pouch volumetry was not feasible. However, every effort was made to maintain blinding of participants and postoperative outcome assessors. Postoperative care protocols and follow-up visit schedules were identical in both groups, minimizing the likelihood that patients could deduce their allocation. Clinical assessors, endoscopists performing follow-up evaluations, and laboratory staff were unaware of the assigned procedure.

### Surgical Technique

All procedures were performed laparoscopically by the same experienced MBS surgical team to minimize inter-operator variability. All patients underwent preoperative ultrasound for gallstone disease, and concomitant cholecystectomy was performed if indicated. Patients were placed in a reverse Trendelenburg position under general anesthesia, and access was obtained via a standard five-trocar technique.

In the standard SG group, gastric division was performed over a 40 Fr calibration bougie (inserted transorally under direct vision to the pylorus) using Echelon Flex Endopath 60-mm linear stapler (Ethicon Endo-Surgery, Cincinnati, Ohio, USA), with green, gold, and blue reloads selected according to tissue thickness. Resection extended from 5 cm proximal to the pylorus up to the angle of His. To reinforce the staple line, running seromuscular sutures were applied using absorbable 3–0 V-Loc™ 180 barbed sutures (Covidien, Mansfield, Massachusetts, USA).

**Fig. 2:**
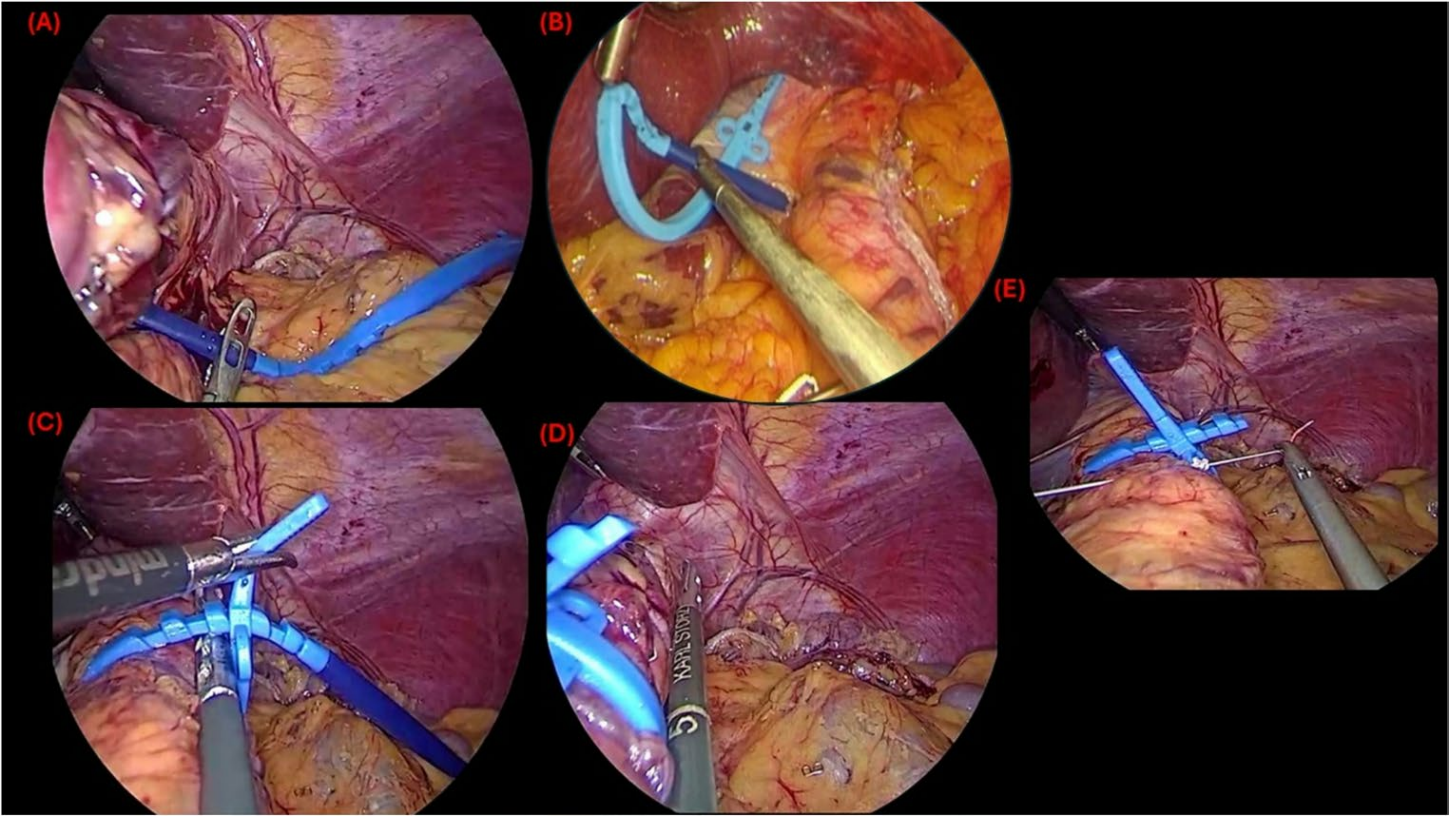
A figure showing the key operative steps of ring-augmented sleeve gastrectomy, Panel (**A**) showing the introduction and positioning of the silicone ring via the pars flaccida window, Panel (**B**) showing creation of a peri-gastric tunnel behind the proximal sleeve (perigastric technique), (**C**) loosening the ring to ensure appropriate non-tensioned laxity by introducing an instrument between the ring and the gastric pouch, (**D**) measurement of the distance from the gastroesophageal junction and the ring, and Panel (**E**) showing fixation of the ring with non-absorbable sutures to prevent migration or slippage

The Ra-SG group followed the same operative steps as SG for sleeve creation. After sleeve formation, a non-adjustable 7.5 cm circumference; 1.75 cm internal diameter MiniMizer Gastric Ring^®^ (Bariatric Solutions GmbH, Stein am Rhein, Switzerland; circumference 7.5 cm, internal diameter 1.75 cm, medical-grade silicone) was introduced and placed approximately 5 cm below the gastroesophageal junction around the proximal gastric sleeve (Fig. 2). The ring was loosely positioned to avoid excessive tension and secured with non-absorbable sutures passed through the ring’s eyelets to prevent migration or slippage [22, 32] (Fig. 2). No intraoperative variation in bougie size or ring specification was permitted, ensuring consistency across all cases. Placement technique followed the manufacturer’s protocol as described earlier [21–23], ensuring atraumatic passage and uniform positioning (Fig. 2). Care was taken to avoid overtightening, which may lead to dysphagia, regurgitation, or ischemic complications [20].

Perioperative management protocols were consistently applied across both cohorts, encompassing the administration of prophylactic antibiotics, thromboprophylaxis, early mobilization strategies, and a gradual dietary progression from liquid to solid foods over six weeks. All participants received uniform postoperative supplementation with vitamins and minerals, along with lifestyle counseling delivered by a multidisciplinary team. Additionally, standardized proton-pump inhibitor (PPI) therapy was prescribed for a duration of six months following surgery. Follow-up evaluations were scheduled at 1, 3, 6, 12, 24, and 36 months postoperative, with allowances for supplementary visits as warranted by clinical necessity.

### Cost

The Institute provided all MiniMizer Gastric Rings and associated hospital costs as part of standard care. Participants did not incur any out-of-pocket expenses related to the device. The manufacturer was not involved in the study design.

### Outcomes

The primary endpoint was the between-group difference in percentage total weight loss (%TWL), percentage excess weight loss (%EWL), and change in body mass index (BMI) at 6, 12, 24, and 36 months postoperatively. %TWL was expressed as the percentage reduction from baseline body weight, and %EWL as the percentage of preoperative excess weight (calculated relative to an ideal body weight corresponding to a BMI of 25 kg/m²) lost at each follow-up interval.

Secondary outcomes comprised the resolution or recurrence of obesity-related diseases, changes in laboratory parameters, and patient-reported outcome measures across all domains. Furthermore, exploratory outcomes included recurrent weight gain (RWG), defined according to the IFSO consensus [33] as late postoperative clinical deterioration characterized by a RWG of more than 30% of the initial weight loss from the nadir or by recurrence/worsening of an obesity-related disease that constituted an indication for surgery. Initial surgical weight loss was calculated as the baseline preoperative weight minus the postoperative nadir weight. RWG% was calculated as 100 × (current weight − nadir weight)/(baseline weight − nadir weight). Nadir weight was defined as the lowest measured postoperative weight within the follow-up window; if multiple minima occurred, the earliest was used. RWG status was evaluated at 12, 24, and 36 months, with event dating based on the first assessment meeting the > 30% criterion.

Other exploratory endpoints included adverse events, reoperations, postoperative endoscopic findings, gastric pouch volume, and revisional MBS procedures. Endoscopic assessment was performed at year 3 post-operatively, and as needed, if the patient was symptomatic, to assess GERD and esophagitis, and was classified according to the LA classification [34]. Reflux symptoms were not systematically collected as predefined outcome measures, as GERD assessment in this trial relied on standardized endoscopic evaluation rather than symptom-based or pharmacologic indices.

Early postoperative complications were graded according to the Clavien–Dindo classification [35], which stratifies surgical complications from grade I (minor deviation from normal postoperative course) to grade V (death). Early complications included leakage and bleeding, while late complications included RWG, the development of gallstones, reflux, or ring-related complications, including migration, slippage, and erosion.

Gastric pouch volume was assessed at the 36-month follow-up using multidetector computed tomography (MDCT) volumetry [36]. All examinations were performed with a 64-detector MDCT scanner (SOMATOM^®^ Perspective, Siemens Medical Solutions, Malvern, PA, USA) using a standardized gastrographic protocol [32]. Patients were instructed to fast for at least four hours prior to imaging, followed by intravenous administration of 40 mg butylscopolamine to minimize gastric peristalsis. Immediately before scanning, participants ingested two to four sachets of effervescent granules (sodium bicarbonate) without water, as tolerated, to achieve gastric distension. All scans adhered to the same institutional gastrographic MDCT protocol described in prior validation studies, with fixed patient positioning, no use of intravenous contrast, and uniform effervescent-based gastric distension; no procedural variations occurred across patients. The MDCT volumetry technique used in this study is consistent with previously published validation studies demonstrating the feasibility and accuracy of CT-based gastric pouch and sleeve volumetric assessment in MBS populations [36–38]. MDCT volumetry was performed at no cost to the patients, with all imaging funded by the university hospital.

Patient-reported outcomes (PROs) included food tolerance (Suter questionnaire [31]) and health-related quality of life (SF-36 [29]). For the SF-36, a validated Arabic version was used [30]. For the Suter questionnaire, Arabic translations were adapted in-house by a bilingual MBS research coordinator through forward–backward translation to ensure linguistic and conceptual equivalence. The validated scoring structure of the Suter FTQ was followed, in which individual item responses are aggregated into a total score; individual food-item responses were not archived separately in the case report forms. The questionnaire was administered in paper format during scheduled clinic visits, with completion supervised by a trained research coordinator who could clarify any items without influencing responses. As part of standard postoperative care, all patients were routinely evaluated by nutritionists and psychiatrists during follow-up visits, and those reporting reduced food tolerance received additional counseling as needed. Baseline assessments were conducted within two weeks prior to surgery, and follow-up questionnaires were conducted at 12, 24, and 36 months during in-person visits or via structured telephone interviews for patients who were unable to attend.

Serial laboratory assessments were performed preoperatively and at 12, 24, and 36 months postoperatively, and included hemoglobin, serum albumin, ferritin, vitamin D, vitamin B12, calcium, fasting glucose, HbA1c, lipid profile, liver enzymes, thyroid function tests, and renal indices.

### Data Variables

PROs were assessed using three validated instruments. The 36-Item Short Form Health Survey (SF-36) evaluates eight health domains: physical functioning, role limitations due to physical health, bodily pain, general health, vitality, social functioning, role limitations due to emotional problems, and mental health [29]. Each domain score ranges from 0 to 100, with higher scores indicating better health status. Physical Component Summary (PCS) and Mental Component Summary (MCS) scores were computed using standard norm-based scoring algorithms. A validated Arabic version was employed [30].

The Suter food tolerance questionnaire comprises three components: ability to consume specific foods (scored 0–27), frequency of vomiting/regurgitation (0–3), and overall tolerance rating (0–10). The total score (0–40) reflects overall food tolerance, with higher scores indicating better tolerance [31].

### Sample Size

The sample size calculation was done using the “pwr” package in R software. We used a medium effect size (Cohen’s d) of 0.5 for the percentage of total weight loss (TWL) or BMI at any follow-up visit between the SG and Ra-SG groups, with a power of 80% and a significance level of 0.05. The effect size of 0.5 corresponded to Cohen’s conventional benchmark for a medium standardized difference. It was selected as a conservative estimate because the existing Ra-SG versus SG literature shows heterogeneous and inconsistent effect estimates across timepoints, precluding a reliable data-driven value. This resulted in a minimum sample size of 64 patients per group at any follow-up visit. Accounting for potential loss to follow-up over the three years, a decision was made to include a total of 219 patients. This sample size was considered sufficient to detect differences not only in the primary weight loss outcome but also in key secondary measures, including the resolution of associated medical problems and gastric pouch volume, without compromising statistical validity.

### Statistical Analysis

Statistical analyses were conducted using R software, version 4.4.2. Basic descriptive statistics, including means, standard deviations, ranges, and proportions, were employed to summarize the baseline characteristics of participants. Comparisons between numerical variables were conducted using t-tests, while categorical variables were analyzed using Chi-square or Fisher’s exact tests, depending on the expected counts in the contingency tables.

Analyses were designed to evaluate the efficacy and safety of SG versus Ra-SG over a three-year period. Two analytical approaches were utilized: a complete data analysis and a complete cases analysis. The complete data analysis employed Generalized Estimating Equations (GEE) to analyze longitudinal data, allowing for the assessment of changes within and between treatment groups across multiple time points while accounting for the correlation of repeated measures within individuals and effectively managing missing data by utilizing all available data points. The complete cases analysis, on the other hand, included only participants who completed all scheduled follow-ups, thereby providing a consistency check against the complete dataset analysis and ensuring the robustness of the findings.

Patterns of missingness were examined across treatment groups for all variables. Missingness followed a monotone pattern driven exclusively by loss to follow-up—patients who missed a given visit were missing all outcomes for that visit (e.g., weight, food tolerance, HRQoL, and laboratory measures). The proportions of missing data were low (< 10%) and highly comparable between Ra-SG and SG at each time point (all *p* > 0.75), with no evidence of differential dropout. Because missing values at 12, 24, and 36 months resulted solely from loss to follow-up, we formally evaluated whether the probability of missingness was related to observed patient characteristics. We fitted separate univariate logistic regression models for missingness at year 1 and at years 2–3 using all available baseline, 6-month, and 1-year clinical and laboratory variables. Missingness was not associated with the treatment group. Although several individual variables showed statistical associations with missingness, these associations were scattered and did not follow a consistent or clinically plausible pattern. Taken together, the findings support that the missing data mechanism was compatible with a Missing at Random (MAR) assumption, under which GEE provides valid inference. Full regression results are available in the Supplementary material.

Outcomes, including weight loss metrics (BMI and Total Weight Loss percentage), quality of life improvements (assessed via SF-36), and food tolerance score, were analyzed using GEE to detect differences from baseline within each group and between groups at each specified time point. All models were fitted using the geepack package in R with an exchangeable working correlation structure (corstr = “exchangeable”). Robust Huber–White sandwich standard errors (the default in “geeglm”) were applied to ensure valid inference even if the working correlation structure was misspecified. A Time × Group interaction term was included in each model (Outcome ~ Time * Group) to evaluate between-group differences at each follow-up interval. Estimated marginal means and time-specific contrasts were obtained using the “emmeans” package. In addition to these contrasts, a global Wald test of the Time × Group interaction was performed for each outcome using joint tests from emmeans to assess overall differences in trajectories across the follow-up period.

For categorical outcomes, such as the persistence or recurrence of associated medical problems, the McNemar test with Bonferroni correction was used to compare differences from baseline within groups at years 1 and 3. Chi-square or Fisher’s exact tests were used to compare between groups at each time point. This approach was suitable for handling binary outcomes derived from the clinical evaluations at each follow-up.

Laboratory parameters were also analyzed using GEE, providing insights into the biochemical and metabolic changes over time. These analyses included evaluations of mean differences from baseline for various biomarkers, such as lipids, glucose, liver enzymes, and nutritional indices. Inter-group comparisons were made to ascertain if there were significant differences in the trajectories of these markers between the SG and Ra-SG groups. Statistical significance was set at *p* < 0.05 for all tests, and all tests were two-tailed.

## Results

### Participant Flow

Initially, 1,900 individuals were assessed for eligibility. After exclusion of 1,681 individuals due to predefined exclusion criteria, including BMI ≤ 50 kg/m² (*n* = 1100), refusal of ring placement (*n* = 122), reflux or proton pump inhibitor (PPI) use or hiatal hernia (*n* = 311), prior surgery (*n* = 92), substance abuse (*n* = 26), pregnancy plans (*n* = 15), uncontrolled psychiatric illnesses (*n* = 6), and cancer diagnoses (*n* = 9), 219 participants were randomized into two groups (Fig. 1). A total of 111 participants received Ra-SG, and 108 received conventional SG. Follow-up was performed at 6 months, 1 year, 2 years, and 3 years, with minimal loss to follow-up. The attrition rate was 8.1% in the Ra-SG group and 9.3% in the conventional SG group by 36 months (Fig. 1).

### Baseline Characteristics

The mean age of participants included in the study was 37.7 ± 10.2 years, with no significant difference between groups (*p* = 0.928) (Table 1). Females constituted 66.7% (*n* = 146) of the participants, with no significant differences between groups (*p* = 0.474). Baseline BMI averaged 53.7 ± 2.8 kg/m² in SG and 53.8 ± 2.9 kg/m² in Ra-SG (*p* = 0.847). Baseline laboratory parameters revealed statistically significant differences between groups for Ferritin (*p* < 0.001), WBC (*p* = 0.045), Creatinine (*p* = 0.014), Free T4 (*p* = 0.014), LDL cholesterol (*p* = 0.009), Calcium (*p* < 0.001), Vitamin D (*p* < 0.001), and Vitamin B12 (*p* < 0.001). The prevalence of diabetes was significantly higher in the Ra-SG group (22.5% vs. 11.1%, *p* = 0.038). All other parameters, including hemoglobin, cholesterol, triglycerides, fasting glucose, and HbA1c, were comparable between groups with no significant differences.

**Table 1:** Baseline demographic, anthropometric, laboratory, associated medical problems, and quality-of-life characteristics of participants randomized to ring-augmented sleeve gastrectomy (Ra-SG) or standard sleeve gastrectomy (SG)

Variable	Total *N* = 219	SG *n* = 108	Ra-SG *n* = 111	*p*
Age	37.7 ± 10.2	37.7 ± 10.2	37.8 ± 10.3	0.928
Sex				
Female	146 (66.7)	69 (63.9)	77 (69.4)	0.474
Male	73 (33.3)	39 (36.1)	34 (30.6)	
Anthropometrics				
Height (m)	1.7 ± 0.1	1.7 ± 0.1	1.7 ± 0.1	0.916
Weight (Kg)	154.5 ± 19.4	154.3 ± 19.4	154.7 ± 19.5	0.871
BMI (kg/m²)	53.8 ± 2.9	53.7 ± 2.8	53.8 ± 2.9	0.847
Lab investigations				
Hemoglobin (g/dl)	12.7 ± 1.5	12.9 ± 1.7	12.6 ± 1.3	0.130
Ferritin (ng/mL)	45.0 ± 49.1	59.9 ± 65.5	30.4 ± 12.8	< 0.001*
White cell count (×10^3^/µL)	7.4 ± 2.4	7.1 ± 2.3	7.7 ± 2.4	0.045*
AST (U/L)	21.2 ± 11.4	21.2 ± 9.3	21.3 ± 13.2	0.930
ALT (U/L)	22.9 ± 13.8	23.0 ± 13.3	22.9 ± 14.4	0.943
Urea (mg/dl)	26.7 ± 8.3	26.4 ± 7.4	27.0 ± 9.2	0.591
Creatinine (mg/dl)	0.8 ± 0.2	0.7 ± 0.2	0.8 ± 0.2	0.014*
INR	1.0 ± 0.1	1.0 ± 0.0	1.0 ± 0.1	0.336
Free T3 (pg/ml)	3.2 ± 0.5	3.2 ± 0.5	3.2 ± 0.6	0.598
Free T4 (ng/dL)	1.2 ± 0.2	1.1 ± 0.2	1.2 ± 0.2	0.014*
TSH (µIU/mL)	2.0 ± 1.0	1.9 ± 0.9	2.1 ± 1.0	0.162
Cholesterol (mg/dL)	180.8 ± 40.4	180.5 ± 39.1	181.1 ± 41.7	0.912
Triglycerides (mg/dl)	147.1 ± 53.9	152.6 ± 50.5	141.7 ± 56.7	0.134
LDL (mg/dL)	101.2 ± 28.3	106.2 ± 26.8	96.3 ± 29.0	0.009*
Albumin (g/dL)	4.5 ± 0.6	4.5 ± 0.6	4.4 ± 0.5	0.209
Calcium (mg/dL)	9.2 ± 0.8	9.1 ± 0.8	9.4 ± 0.7	< 0.001*
Vitamin D (ng/mL)	27.8 ± 12.0	31.1 ± 11.7	24.6 ± 11.5	< 0.001*
Vitamin B12 (pg/ml)	524.5 ± 290.8	604.9 ± 367.3	446.2 ± 154.3	< 0.001*
Fasting glucose (mg/dL)	96.1 ± 22.0	96.1 ± 23.9	96.1 ± 20.1	0.993
HbA1c %	5.3 ± 0.6	5.3 ± 0.6	5.3 ± 0.5	0.665
Preoperative radiology				
Gall stones	22 (10.0)	9 (8.3)	13 (11.7)	0.544
Associated medical illnesses				
Hypertension	68 (31.1)	32 (29.6)	36 (32.4)	0.763
Diabetic	37 (16.9)	12 (11.1)	25 (22.5)	0.038*
Obstructive Sleep Apnea	18 (8.2)	8 (7.4)	10 (9.0)	0.853
Dyslipidemia	68 (31.1)	30 (27.8)	38 (34.2)	0.375
Osteoarthritis	54 (24.7)	30 (27.8)	24 (21.6)	0.368
History Cardiac Conditions	14 (6.4)	10 (9.3)	4 (3.6)	0.151
SF-36 (score 0–100)				
Physical Functioning	41.1 ± 12.1	41.3 ± 11.8	41.0 ± 12.5	0.833
Role Physical	40.3 ± 10.8	39.9 ± 10.3	40.7 ± 11.2	0.574
Pain	38.4 ± 11.8	36.6 ± 11.5	40.2 ± 11.8	0.024*
General Health	40.8 ± 11.1	40.8 ± 11.0	40.9 ± 11.2	0.946
Social Functioning	39.5 ± 12.1	39.8 ± 12.0	39.1 ± 12.2	0.648
Role Emotional	40.2 ± 11.9	38.8 ± 12.3	41.6 ± 11.3	0.083
Vitality	39.1 ± 11.1	39.3 ± 10.8	38.8 ± 11.4	0.715
Mental Health	40.3 ± 11.0	39.9 ± 11.3	40.7 ± 10.9	0.595
Physical component summary	43.6 ± 12.3	42.6 ± 12.5	44.7 ± 12.1	0.210
Mental component summary	41.2 ± 13.2	42.3 ± 13.5	40.1 ± 12.9	0.229

Values are presented as mean ± standard deviation for continuous variables and n (%) for categorical variables. *p*-values represent between-group comparisons at baseline; values marked with an asterisk (*) indicate statistical significance at *p *< 0.05. All patients with gallstones haveundergone concomitant cholecystectomy during the metabolic procedure performed. *BMI* bodymass index; *AST *aspartate aminotransferase; *ALT* alanine aminotransferase; *INR* international normalized ratio; *FT3 *free triiodothyronine; *FT4* free thyroxine; *TSH* thyroid-stimulating hormone; *LDL* low-density lipoprotein; *HbA1c* glycated hemoglobin; *SF-36* 36-Item Short Form Health Survey. Higher SF-36 scores indicate better health-related quality of life

### Weight Loss Outcomes

**Fig. 3:**
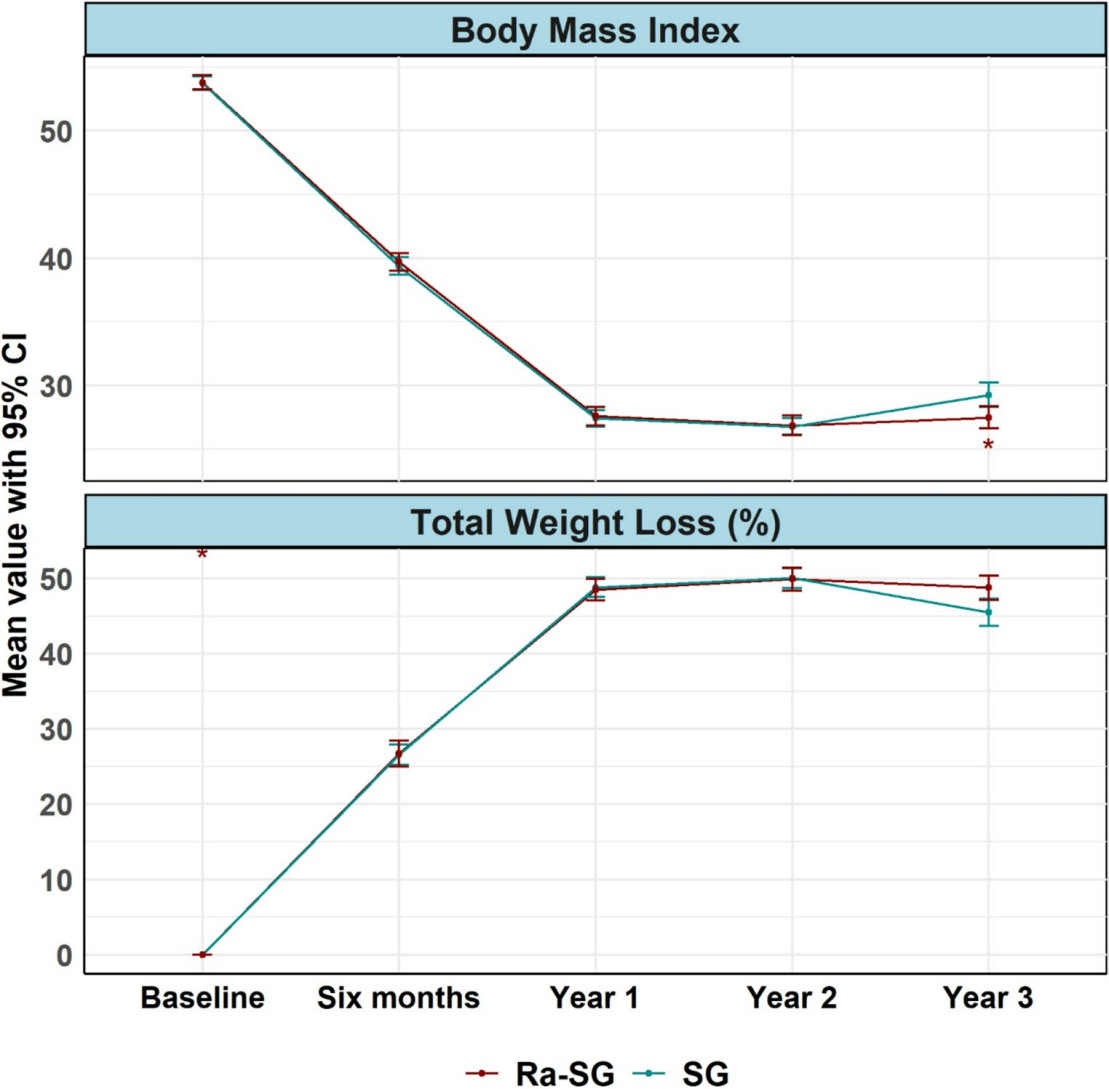
Longitudinal outcomes of BMI and total weight loss over three years. This figure displays the trends in Body Mass Index (BMI) and Total Weight Loss percentages (TWL%) for patients undergoing Sleeve Gastrectomy (SG) and Ring-augmented Sleeve Gastrectomy (Ra-SG) over a three-year period. The plots illustrate significant weight loss in both groups with progressive increases in TWL percentages. Notably, by Year 3, the Ra-SG group demonstrates significantly better maintenance of weight loss, as evidenced by lower BMI values and higher TWL percentages, compared to the LSG group. Mean differences with 95% confidence intervals at Year 3 highlight the statistical significance of these outcomes

Both groups demonstrated substantial weight reduction after surgery, with comparable outcomes across most postoperative intervals. A significantly greater weight loss was observed in the Ra-SG group only at year 3 (Fig. 3). At 6 months, mean %TWL was 26.8 ± 9.5% in Ra-SG vs. 26.7 ± 6.7% in SG (*p* = 0.975), increasing to 48.6 ± 7.7% vs. 49.1 ± 6.6% at 12 months (*p* = 0.632) and remained stable at 24 months (49.9 ± 7.7% vs. 50.1 ± 6.6%; *p* = 0.875). By 36 months, %TWL declined slightly in both cohorts but was significantly greater in the Ra-SG (48.8 ± 8.3% vs. 45.5 ± 9.0%; *p* = 0.008).

Baseline BMI was nearly identical (Ra-SG 53.8 ± 2.9 kg/m², SG 53.8 ± 2.8 kg/m²; *p* = 0.980). At 6 months, BMI fell to 39.7 ± 3.7 kg/m² (Ra-SG) and 39.4 ± 3.5 kg/m² (SG; between-group MD = − 0.35, 95% CI − 1.35 to 0.65; *p* = 0.495). At 12 months it reached 27.6 ± 3.9 vs. 27.3 ± 3.3 kg/m² (MD = − 0.26; 95% CI − 1.26 to 0.73; *p* = 0.607), at 24 months 26.9 ± 3.9 vs. 26.8 ± 3.2 kg/m² (MD = − 0.08; 95% CI − 1.08 to 0.91; *p* = 0.873), and at 36 months 27.5 ± 4.4 vs. 29.3 ± 4.7 kg/m² (MD = 1.77; 95% CI 0.52–3.03; *p* = 0.006) (Fig. 3). BMI reduction plateaued after the first postoperative year, but Ra-SG maintained a significantly lower mean BMI at 36 months (Supplementary File 1; Tables S1 and S2).

Global Wald tests of the Time × Group interaction were not statistically significant for %TWL (*p* = 0.122) or BMI (*p* = 0.086), indicating that overall longitudinal trajectories were similar between groups, and consistent with the finding that between-group differences emerged only at the 36-month assessment.

### Recurrent Weight Gain and Pouch Volumetry

**Fig. 4:**
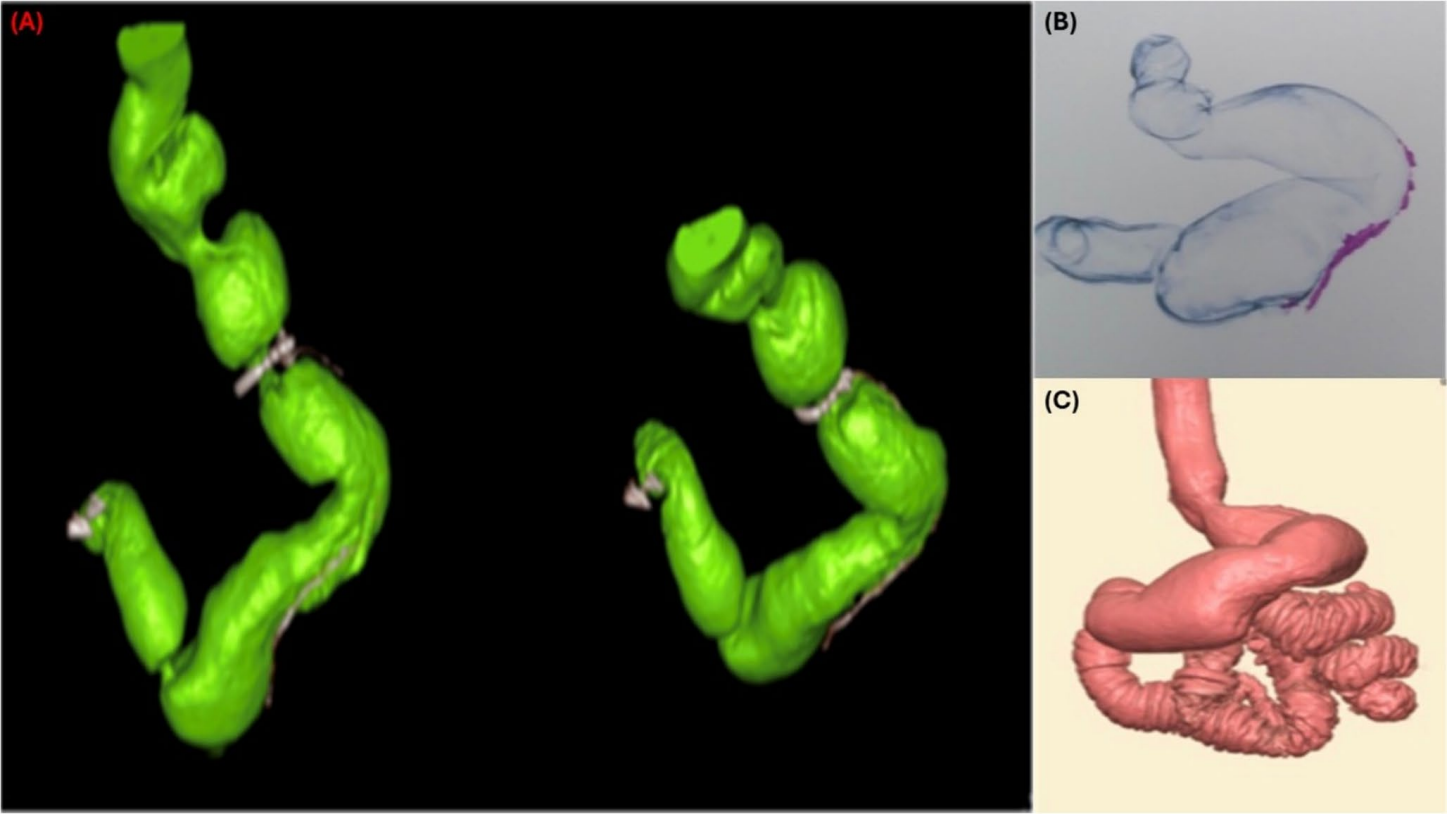
Postoperative 3-D CT volumetry of the gastric sleeve at 36-month follow-up. Surface-rendered reconstructions after oral contrast with measured pouch volumes: (**A**) ring-augmented sleeve gastrectomy (Ra-SG), 120 mL (two views of the same case; ring visible; small sliding hiatal hernia), (**B**) standard sleeve gastrectomy (SG), 160 mL, (**C**) SG, 180 mL

At 36 months, mean absolute RWG from nadir was significantly lower in the Ra-SG group compared with the SG group (2.7 ± 6.9 kg vs. 7.6 ± 10.2 kg, *p* < 0.001) (Table 2). When expressed as a percentage of the maximum weight lost, recurrent weight gain percentage (RWG%) was 3.4 ± 7.6% in Ra-SG vs. 9.8 ± 12.9% in SG (*p* < 0.001) (Table 2). Clinically significant RWG, defined as ≥ 30% RWG from nadir, occurred in 5.9% of Ra-SG patients compared with 16.3% in SG (*p* = 0.033) (Table 2). Gastric pouch volumetry at year 3 demonstrated a smaller mean pouch volume in the Ra-SG group (160.1 ± 8.9 mL) than in the SG group (194.2 ± 10.3 mL, *p* < 0.001) (Table 2) (Fig. 4).

**Table 2:** Operative characteristics, postoperative complications, follow-up endoscopic findings, revisional procedures, gastric pouch volumetry, recurrent weight gain, and relapse of obesity-associated diseases in the standard sleeve gastrectomy (SG) and ring-augmented sleeve gastrectomy (Ra-SG) groups

Variable	SG *n* = 108	Ra-SG *n* = 111	*p*
Operative time (min)	41.2 ± 7.3	41.8 ± 7.8	0.600
Early complications			
Leak	1 (0.9)	0 (0.0)	0.493
Bleeding	1 (0.9)	1 (0.9)	1.000
Late complication			
Calculous Cholecystitis	6 (5.6)	11 (9.9)	0.341
Removal of the ring due to intolerance		2 (1.8)	
Clavien-Dindo classes			
Clavien-Dindo I	10 (9.3)	16 (14.4)	0.332
Clavien-Dindo II	1 (0.9)	1 (0.9)	1.000
Clavien-Dindo III	1 (0.9)	0 (0.0)	0.989
Endoscopic Findings in Year 3	*n* = 98	*n* = 102	
Free	74 (75.5)	77 (75.5)	1.000
GERD	17 (17.3)	20 (19.6)	0.818
Reflux grade C	2 (2.0)	1 (1.0)	0.616
Hiatal hernia	4 (4.1)	5 (4.9)	1.000
Constriction at the incisura	3 (3.1)	0 (0.0)	0.116
Follow-up	*n* = 98	*n* = 102	
* Revisional surgery*	8 (7.2)	2 (2)	0.055
None	90 (91.8)	100 (98.0)	0.053
OAGB due to recurrent weight gain (RWG)	2 (2.0)	0 (0.0)	
RYGB due to reflux	0 (0.0)	1 (1.0)	
RYGB due to RWG	4 (4.1)	1 (1.0)	
RYGB due to RWG and reflux	2 (2.0)	0 (0.0)	
* Pouch volumetry (mL)*	194.2 ± 10.3	160.1 ± 8.9	< 0.001*
* Recurrent Weight Gain (RWG)*			
Weight gained (Kg)	7.6 ± 10.2	2.7 ± 6.9	< 0.001*
RWG percentage from the maximum weight lost (%)	9.8 ± 12.9	3.4 ± 7.6	< 0.001*
RWG ≥ 30% (n (%))	16 (16.3)	6 (5.9)	0.033*
Disease relapse after initial resolution at year 1:			
Cardiac Conditions	3 (3.1)	0 (0.0)	0.116
Diabetes	2 (2.0)	1 (1.0)	0.616
Dyslipidemia	7 (7.1)	0 (0.0)	0.006*
Hypertension	7 (7.1)	0 (0.0)	0.006*
Osteoarthritis	6 (6.1)	2 (2.0)	0.164
Obstructive sleep apnea	2 (2.0)	0 (0.0)	0.239
Worsening of any associated medical problems that was initially resolved	15 (15.3)	3 (2.9)	0.003*

Values are presented as mean ± standard deviation for continuous variables and n (%) for categorical variables. *p*-values represent between-group comparisons; values marked with an asterisk (*) indicate statistical significance at *p *< 0.05. Patients who developed gallstones post-operatively are calculated from the whole cohort sample, including those who underwent concomitant cholecystectomy during the procedure. Clavien–Dindo classification: Grade I, minor deviation from normal postoperative course; Grade II, pharmacological treatment; Grade III, surgical, endoscopic, or radiologic intervention. *GERD* gastroesophageal reflux disease; *OAGB* one-anastomosis gastric bypass; *RYGB *Roux-en-Y gastric bypass; *RWG* recurrent weight gain

### Resolution and Relapse of Obesity-related Diseases

Initial postoperative resolution of obesity associated diseases at year 1 was high in both groups (Supplementary File 1, Fig. S1). Between years 1 and 3, relapse rates were consistently lower in Ra-SG. No cases of hypertension or dyslipidemia relapse occurred in Ra-SG, compared with 7.1% for each in SG (both *p* = 0.006). Relapse of type 2 diabetes was low in both groups (SG 2.0%, Ra-SG 1.0%, *p* = 0.616), as was relapse of cardiac disease (SG 3.1%, Ra-SG 0%, *p* = 0.116), osteoarthritis (SG 6.1%, Ra-SG 2.0%, *p* = 0.164), and obstructive sleep apnea (SG 2.0%, Ra-SG 0%, *p* = 0.239). The proportion of patients experiencing relapse of any obesity related disease was significantly higher after SG (15.3%) than after Ra-SG (2.9%, *p* = 0.003) (Table 2). Supplementary File 1; Table S7 shows within-group analyses confirming significant improvements from baseline in both arms, with greater durability in Ra-SG.

### Postoperative Complications and Revisional Surgery

Mean operative time was similar between SG and Ra-SG (41.2 ± 7.3 min vs. 41.8 ± 7.8 min, *p* = 0.600). Early postoperative complications (≤ 30 days) were rare in both groups, with one leak in SG (0.9%) and no leaks in Ra-SG, and one bleeding event in each group (0.9%), with no significant differences. Late complications included chronic calculous cholecystitis in 5.6% of SG patients and 9.9% of Ra-SG patients (*p* = 0.341) (Table 2).

Early postoperative morbidity was low in both groups. Clavien–Dindo grade I events occurred in 9.3% of SG and 14.4% of Ra-SG patients (*p* = 0.332); grade II in 0.9% of each group (*p* = 1.000); grade IIIa was absent in both arms; and grade IIIb occurred in 0.9% after SG versus none after Ra-SG (*p* = 0.989). No grade IV–V complications were observed. Overall, any early Clavien–Dindo event occurred in 11.1% of SG and 15.3% of Ra-SG (*p* = 0.473). The single early leak in the SG arm required combined endoscopic and radiologic intervention under general anesthesia and was therefore classified as CD IIIb.

Late complications were analyzed separately. In the SG group, by year 3, revisional surgery included one conversion to RYGB for reflux, four conversions to RYGB for recurrent weight gain (RWG), two conversions to RYGB for combined RWG and reflux, and two conversions to OAGB for RWG. In the Ra-SG group, two patients required conversion to RYGB in the second postoperative year, one for reflux and one for RWG (Table 2). Two additional Ra-SG patients underwent ring removal for intolerance in the second year (Table 2).

### Endoscopic Findings

Follow-up EGD was performed at year 3 in 98 SG patients and 102 Ra-SG patients (Table 2). The proportion of patients with a normal postoperative EGD was identical in both groups (75.5%). Gastroesophageal reflux disease (GERD) was observed in 17.3% of SG and 19.6% of Ra-SG patients (*p* = 0.818). Esophagitis grade C was infrequent, occurring in 2.0% of SG and 1.0% of Ra-SG (*p* = 0.616). Hiatal hernia was detected in 4.1% of SG and 4.9% of Ra-SG (*p* = 1.000). Constriction at the incisura angularis was found in 3.1% of SG and in none of the Ra-SG patients (*p* = 0.116).

### Laboratory Outcomes

Across follow-up, both groups demonstrated significant improvements in nutritional, metabolic, and endocrine markers. At 36 months, between-group differences in ferritin, 25-hydroxy-vitamin D, vitamin B12, and calcium were small and not statistically significant after Bonferroni adjustment; mean values were similar (SG vs. Ra-SG at 36 months: vitamin D 33.7 ± 9.6 vs. 32.2 ± 10.1 ng/mL (Supplementary File 1; Fig. S2); vitamin B12 591.5 ± 159.1 vs. 543.8 ± 163.1 pg/mL; calcium 8.9 ± 0.5 vs. 8.8 ± 0.5 mg/dL; ferritin 74.4 ± 41.8 vs. 71.8 ± 45.2 ng/mL (Supplementary File 1; Tables S8 and S9, Fig. S3). Intermittent, time-point–specific differences favoring Ra-SG were observed for LDL-cholesterol (baseline 96.3 vs. 106.2 mg/dL; year 2: 70.6 vs. 64.5 mg/dL, *p* = 0.015) and fasting glucose (lower at 6 and 24 months, *p* < 0.001), but these effects were not consistent across all visits and did not persist as significant between-group differences at 36 months after multiplicity correction. Time-specific differences also appeared for triglycerides, cholesterol, AST, creatinine, and urea, but did not persist after multiplicity correction.

### Patient-reported Outcomes

**Fig. 5:**
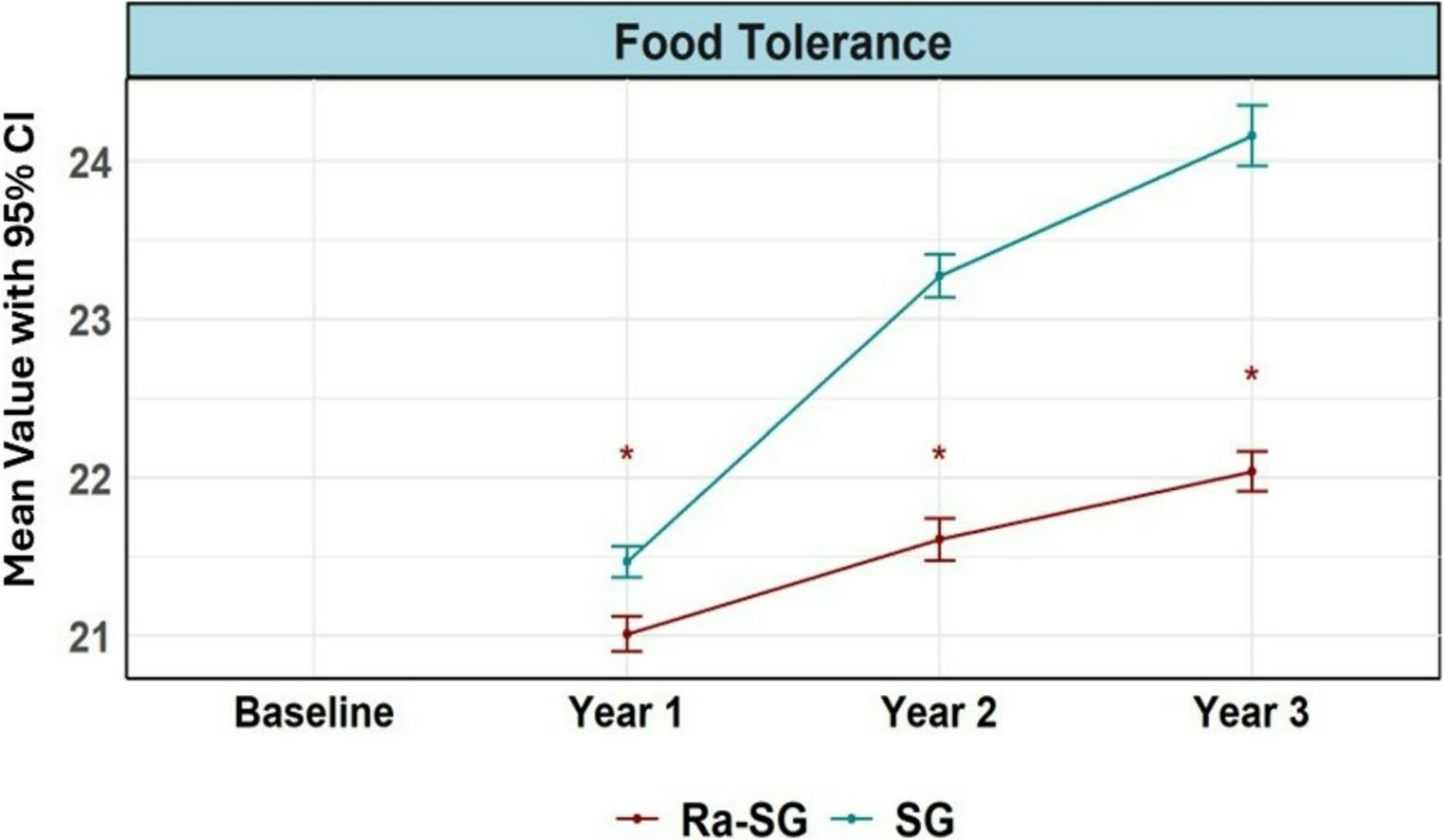
Progression of food tolerance over three years post-surgery. This figure illustrates the changes in food tolerance for patients undergoing Sleeve Gastrectomy (SG) and Ring-augmented Sleeve Gastrectomy (Ra-SG) over a three-year period. Food tolerance improved more significantly in the SG group compared to the Ra-SG group, with SG patients experiencing notably greater enhancements in their ability to tolerate a variety of foods over time

Across the cohort, health-related quality of life (HRQoL; SF-36) improved significantly from baseline through 36 months in both arms, with no sustained between-group differences at year 3. These findings indicate that HRQoL gains were comparable after SG and Ra-SG (Supplementary Tables S3–S4, Supplementary Fig. S4). Food tolerance (Suter questionnaire) improved over time in both groups but remained consistently better after standard SG at every assessed time point (12, 24, and 36 months), with all between-group comparisons favoring SG (all *p* < 0.05) (Fig. 5). This pattern was supported by the global Wald test of the Time × Group interaction, which was highly significant (*p* < 0.001), indicating consistently superior food tolerance in the SG group across the entire follow-up period.

## Discussion

### Weight Loss Outcomes and Durability

In our cohort, Ra-SG achieved superior 3-year weight loss outcomes compared with standard SG, with a mean percentage of total weight loss (TWL) of 48.8% versus 45.5% (*p* = 0.008). Consistently, mean pouch volume was smaller in Ra-SG (160.1 ± 8.9 mL) than in SG (194.2 ± 10.3 mL, *p* < 0.001). These findings align with Lemmens et al., who demonstrated in patients with severe obesity (BMI ≥ 50) that mean %EWL was significantly higher after Ra-SG (78.3 ± 1.6) compared with SG (61.6 ± 24.6), with 35.2% of SG patients failing to reach %EWL ≥ 50% versus none in the Ra-SG group [39]. Similarly, Fink et al. reported a 9% higher percentage of excess weight loss (EWL) at five years with Ra-SG, and Chaouch et al. observed an 8–10% greater rate of EWL at 1–3 years [40]. These converging results emphasize that Ra-SG offers not only superior weight loss in mixed cohorts but also clear advantages in patients with a BMI > 50 kg/m², where SG typically underperforms [11–15].

### Recurrent Weight Gain (RWG)

The durability of weight loss remains a central concern, as up to 30% of SG patients experience substantial weight regain within 5–7 years [7]. Our trial showed clinically significant RWG (≥ 30% of maximum loss) in 16.3% of SG patients, compared to only 5.9% after Ra-SG (absolute risk difference, − 10.4%; 95% CI, − 19.6% to − 1.2%; *p* = 0.033). Prior evidence supports this “anti-regain” effect: Lemmens et al. found significant RWG in 19.6% of SG versus 2% of Ra-SG patients at five years [39], Hany et al. reported RWG > 10% from nadir in 10.6% vs. 3.1% over four years [32], and Fink et al. noted conversion to bypass for suboptimal clinical responsewas nearly twice as frequent after SG (20% vs. 11.9%) [24].

The identification of patient-level predictors of RWG is clinically relevant when considering which individuals may benefit most from ring augmentation. Prior studies have shown that higher preoperative BMI, older age, obstructive sleep apnea, hepatic steatosis, emotional eating patterns, and early insufficient weight loss are among the strongest predictors of long-term RWG after SG [16–19]. These risk factors closely reflect the phenotype of our cohort (BMI > 50 kg/m²) and reinforce the rationale for evaluating whether the anatomical durability offered by Ra-SG may provide additional protection against relapse in individuals inherently predisposed to RWG.

Mechanistically, the silastic ring maintains a fixed outlet 4–5 cm below the gastroesophageal junction, limiting reservoir expansion and slowing emptying [39, 41]. Radiological data confirm smaller volumes years after Ra-SG [42], and delayed gastric transit may activate the “ileal brake,” prolonging satiety [39]. Consistent with these mechanisms, our cohort demonstrated significantly smaller pouch volumes at year 3 after Ra-SG. Together, these mechanisms explain how Ra-SG augments both the magnitude and durability of weight loss in severe obesity.

Importantly, our trial addresses a critical gap by providing the first randomized evidence in a homogeneous cohort with a BMI > 50 kg/m². Previous studies in mixed populations suggested ring augmentation improved %EWL and RWG by 5–10% [39–42], but their applicability to patients with severe obesity was uncertain. By demonstrating that Ra-SG maintains significantly greater %TWL at three years and reduces RWG compared with SG, our study provides the most robust evidence to date that ring augmentation enhances more sustained outcomes in patients with severe obesity.

### Food Tolerance

The principal trade-off for improved weight maintenance is reduced food tolerance. In our series, Ra-SG patients reported more difficulty eating, consistent with higher rates of regurgitation and dysphagia reported elsewhere. Fink et al. noted weekly postprandial regurgitation in 59% of Ra-SG versus 23% of SG patients at five years [24], and Hany et al. found significantly worse mid-term food tolerance scores, despite similar early vomiting rates [32]. In our trial, food tolerance scores were consistently higher after SG at all follow-ups (all *p* < 0.05), confirming a persistent difference beyond the early postoperative phase [32]. The fixed ring likely causes these symptoms by creating a smaller effective outlet; rapid eating or large boluses can transiently obstruct passage, provoking regurgitation.

Most intolerance is functional and improves with adaptation. Patients learn to chew thoroughly and eat slowly. Lemmens et al. observed that many adapted without dissatisfaction, perhaps to preserve the perceived weight benefit [39]. Objective data show no significant early postoperative difference in vomiting or GERD between Ra-SG and SG, but consistently worse long-term food tolerance with the ring [23, 32, 42]. Mild regurgitation or the urge to vomit can occur if patients overeat, making preoperative counseling essential. Suitable candidates must understand the balance between maintaining weight and the likelihood of adopting stricter lifelong dietary habits, which may occasionally lead to discomfort. With careful selection and education, most individuals find these symptoms manageable; however, reduced food tolerance remains a notable drawback of ring augmentation, occasionally contributing to patient dissatisfaction and, in rare cases, necessitating ring removal (1.8% in our cohort) [40, 43].

Although FTQ scores were analyzed longitudinally, the questionnaire’s composite structure and the absence of archived item-level responses did not allow us to identify individual adaptation trajectories or determine whether patients with early intolerance later improved. Future studies incorporating symptom-specific or item-level longitudinal tracking could more precisely characterize the dynamics of eating adaptation after ring augmentation.

### Revisions, Complications, and Nutritional Parameters

The safety profile of Ra-SG must be clearly distinguished from that of the historically adjustable gastric band, which carried long-term reoperation rates exceeding 30% due to slippage, erosion, and migration [44, 45]. In contrast, the non-adjustable rings used in Ra-SG have demonstrated relatively low device-related complication rates [22–24, 32, 40, 42],], although no direct head-to-head comparisons are available. In the prospective series by Lemmens et al., 3.1% of patients required ring enlargement, and only 1% underwent ring removal within five years [39]. Across the literature, revision rates directly attributable to the ring are reported between 3% and 11%, which remains modest compared with failure-driven reoperation rates after standard SG, where conversion to bypass for RWG approaches 30% [7, 20, 24, 42]. At the three-year mark, the cumulative reoperation rate following Ra-SG was 3.6%, comprising 2.0% conversions to gastric bypass and 1.8% instances of ring removals. In contrast, the reoperation rate after standard SG was 7.2%. Thus, while some ring-related revisions occur, Ra-SG may ultimately reduce the overall burden of reoperation by lowering the incidence of failure and recurrence.

General complication rates are comparable between Ra-SG and SG. Operative time and early postoperative events, including bleeding, leaks, and infection, do not differ significantly with ring placement; However, endoscopy-defined GERD at year 3 was 17.3% after SG and 19.6% after Ra-SG (*p* = 0.818), with esophagitis (Los Angeles grade C) in 2.0% after SG vs. 1.0% after Ra-SG (*p* = 0.616). These estimates are lower than symptom-based rates reported elsewhere; for example, a 5-year randomized trial reported weekly reflux symptoms in approximately 27% in each arm [24], consistent with Chaouch et al.’s meta-analysis showing no significant difference in de novo GERD between Ra-SG and SG (OR 0.65, 95% CI 0.34–1.26, *p* = 0.20) [40].

Our methodology explains these discrepancies: we used routine EGD at 36 months, which is specific for erosive disease but insensitive to non-erosive reflux. A substantial proportion of symptomatic GERD patients have normal EGD findings, mainly when assessed while on acid suppression [46, 47]. In contrast, ambulatory pH-impedance monitoring off therapy is the most sensitive objective test for pathologic reflux in “unproven GERD,” with consensus diagnostic thresholds and adjunct impedance metrics when endoscopy is normal [48]. Consistent with these principles, our EGD-defined rates (year-3 GERD: 17.3% SG vs. 19.6% Ra-SG; grade C esophagitis: 2.0% vs. 1.0%) likely underestimate non-erosive reflux. Combined with prior RCT and meta-analysis [24, 40], these findings support our conclusion that ring augmentation does not worsen reflux versus SG across assessment approaches.

Nutritional outcomes have been less extensively studied. Both Ra-SG and SG are primarily restrictive procedures and follow similar supplementation protocols, so major long-term differences are not expected. Some studies have reported isolated differences: for example, Fink et al. observed higher serum vitamin D levels in Ra-SG patients at 6 months and improved vitamin B12 status at 2 years compared with SG [24]. However, these findings have not been consistently reproduced and may reflect short-term variability rather than a sustained nutritional advantage. Consistent with our findings, laboratory profiles indicated sustained nutritional adequacy in both arms, with no consistent between-group advantage observed at 36 months. Future multicenter trials with standardized nutritional monitoring and reflux phenotyping (symptoms, EGD, and pH-impedance) are warranted to confirm long-term safety and refine patient selection.

### Metabolic Improvements and Recurrence of Obesity-Related Diseases

Both Ra-SG and standard SG result in marked improvements in obesity-related problems, including type 2 diabetes, hypertension, dyslipidemia, sleep apnea, and osteoarthritis. However, differences may emerge in the durability of these effects. Fink et al. suggested that patients undergoing SG experience higher recurrence rates of hypertension and dyslipidemia compared with those treated with Ra-SG [24], aligning with our results. This pattern is likely attributable to the more durable weight control achieved with the ring, as RWG is a well-recognized driver of cardiometabolic relapse [41]. Mechanistically, poor lipid control has been shown to correlate with secondary hypertension, providing a plausible link between recurrent dyslipidemia and blood pressure relapse in this context [49–53].

Nevertheless, randomized and meta-analytic evidence to date has not demonstrated statistically significant differences in remission rates of associated medical problems between Ra-SG and SG at medium-term follow-up [24, 40, 42]. In Fink et al.’s 5-year randomized trial, diabetes remission rates were comparable (64% in Ra-SG vs. 67% in SG), as were reductions in antihypertensive use [24]. Chaouch et al.’s meta-analysis also found no clear metabolic advantage of the ring beyond weight outcomes [40].

Taken together, these findings suggest that both procedures substantially improve metabolic health, but Ra-SG may indirectly help reduce the recurrence of hypertension and dyslipidemia by sustaining weight loss. The ring does not appear to exert independent metabolic effects, and its benefits in this domain are therefore secondary to its impact on long-term weight control.

### Health-Related Quality of Life (HRQoL)

Both SG and Ra-SG are associated with significant improvements in HRQoL, largely reflecting the benefits of sustained weight loss. Prospective cohorts consistently demonstrate substantial gains across physical, social, and psychological domains within the first 1–2 years after surgery, as assessed by validated instruments such as SF-36 and BQL, where lower HRQoL has been linked to RWG and symptomatic reflux [54, 55]. Similar improvements have been reported using BAROS/Moorehead-Ardelt scoring [56].

Importantly, no consistent differences have been observed between Ra-SG and SG [24, 40], and in our cohort, HRQoL at three years was comparable between groups. These findings also suggest that the ring does not introduce new quality-of-life impairments beyond those seen with SG, reinforcing the interpretation that HRQoL improvements are primarily driven by weight loss rather than attributable to the ring itself. This underscores that the main contribution of ring augmentation lies in enhancing the durability of weight control rather than conferring additional quality-of-life benefits.

### Strengths and Limitations

The principal strengths of this trial include its prospective, randomized design, adequate sample size, and three-year follow-up with low attrition, which together enhance the reliability of weight-loss durability estimates. The use of standardized outcome measures for associated medical problems, HRQoL, and food tolerance further supports internal validity. The focus on patients with BMI > 50 kg/m² addresses an underrepresented, high-risk subgroup, while the volumetric and patient-reported endpoints add granularity beyond weight trajectories alone.

Nevertheless, Several limitations merit consideration. The single-center design and conduct by one surgical team may limit external generalizability, particularly with respect to technical nuances and perioperative protocols. Although the trial was adequately powered to detect weight outcomes, it was not sufficiently powered to detect more subtle differences in nutritional indices or health-related quality of life domains. The use of simple randomization without stratification led to baseline imbalances in some laboratory measures; while randomization preserves unbiasedness on average, the potential influence of these differences on selected secondary outcomes cannot be excluded. Because ferritin, calcium, vitamin D, and B12 differed modestly between groups at baseline, these imbalances should be acknowledged when interpreting secondary biochemical outcomes. Although such micronutrient variations are unlikely to materially affect the primary weight-loss endpoints, their presence warrants cautious interpretation of longitudinal laboratory comparisons.

In addition, while MDCT-based gastric sleeve volumetry has been used and validated in prior MBS literature, there remains limited large-scale validation specifically in ring-augmented sleeve gastrectomy populations. Our protocol builds on established methods, but the applicability of MDCT volumetry to ring-augmented constructs warrants further multicenter evaluation.

Secondly, our cohort included patients with a BMI > 50 kg/m²; however, there were no patients included within the trial above a BMI of 59.7 kg/m². While this selection reduces bias, it does not generalize the data to all patients with severe obesity.

Furthermore, GERD assessment relied exclusively on endoscopic evaluation at 36 months, without systematic collection of reflux symptoms or longitudinal PPI usage. As EGD underdetects non-erosive reflux, our estimates likely underrepresent the true prevalence of symptomatic or acid-suppressed reflux across both groups. This limitation should be considered when interpreting the reflux-related findings.

Finally, although the Suter FTQ was administered prospectively and scored according to its validated structure, individual food-item responses were not archived separately, which precluded component-level analysis; only domain-level and total scores were available. The 36-month follow-up horizon, although longer than most SG trials, is still insufficient to fully assess late mechanical complications, including rare but clinically meaningful events such as ring erosion, delayed migration, or late device-related intolerance, or to evaluate long-term metabolic relapse. The single-center, single-surgeon design also raises the possibility of procedure-specific or device-handling bias that may not fully reflect practice variability across programs. Future studies should include multicenter trials with extended follow-up and integration with registry-based, patient-centered outcomes to validate and extend these findings. Our center is planning to publish the longer-term results for this cohort.

### Clinical Implications and Recommendations for Future Research

This trial shows that Ra-SG enhances mid-term weight loss durability compared to standard SG, with similar perioperative safety and GERD outcomes but lower food tolerance in some patients. These findings support the role of Ra-SG as a selective “anti-regain” strategy, particularly for patients with severe obesity. Careful preoperative counseling is essential to balance the benefits of sustained weight control against the likelihood of stricter lifelong dietary habits and occasional regurgitation.

Future research should move beyond single-center proof-of-concept trials toward large-scale, multicenter studies with follow-up beyond five years. Such trials should compare Ra-SG not only to SG but also to other ring-reinforced procedures, incorporating comprehensive nutritional assessments and standardized patient-reported outcomes. Multicenter designs will be essential to assess reproducibility across different surgical teams and devices, while extended follow-up is required to characterize very late ring-related adverse events, including erosion or delayed migration. Cost-effectiveness analyses are also warranted to determine whether the reduction in recurrent weight gain offsets the added procedural complexity and device cost. Additionally, future studies should incorporate physiological GERD testing, such as pH-impedance monitoring in selected subsets, to complement endoscopic evaluation and better define reflux phenotypes after Ra-SG. Registry integration will be crucial to assess late complications, durability of weight control, and quality-of-life trade-offs at a population level.

## Conclusion

Ra-SG achieved statistically greater weight-loss durability at a mid-term follow-up of 36 months than standard SG, with a lower incidence of clinically significant RWG and smaller gastric pouch volumes at three years. Safety was comparable between procedures, with rare early complications in both groups. By three years, re-operation was less frequent after Ra-SG; ring removal was uncommon and was counted as a reoperation. Endoscopy-defined GERD at three years was similar between groups, including low rates of high-grade esophagitis, whereas food tolerance consistently favored standard SG at all follow-ups. Overall, Ra-SG is a reasonable option for patients at high risk of RWG and with a BMI > 50 kg/m², provided that counseling addresses stricter eating behavior and the minor possibility of ring-related reoperation. Confirmation in multicenter cohorts with longer follow-up is warranted.

## Supplementary Information

Below is the link to the electronic supplementary material.

Supplementary Material 1

## Data Availability

The datasets generated and analyzed during this study are available from the corresponding author upon reasonable request. The full R analysis scripts used in the study were provided to the reviewers as confidential supplementary material during peer review and remain available from the corresponding author upon reasonable request, in accordance with institutional and ethical regulations.

## References

[1] Rubino F, Cummings DE, Eckel RH, Cohen RV, Wilding JPH, Brown WA, Stanford FC, Batterham RL, Farooqi IS, Farpour-Lambert NJ, et al. Definition and diagnostic criteria of clinical obesity. Lancet Diabetes Endocrinol. 2025;13(3):221–62. 10.1016/S2213-8587(24)00316-4. 39824205 10.1016/S2213-8587(24)00316-4 PMC11870235

[2] Dicker D, Sagy YW, Ramot N, Battat E, Greenland P, Arbel R, et al. Bariatric metabolic surgery vs glucagon-like peptide-1 receptor agonists and mortality. JAMA Netw Open. 2024;7(6):e2415392-2415392. 10.1001/jamanetworkopen.2024.15392. 38848064 10.1001/jamanetworkopen.2024.15392 PMC11161844

[3] Wu H, Yang W, Guo T, Cai X, Ji L. Trajectory of the body weight after drug discontinuation in the treatment of anti-obesity medications. BMC Med. 2025;23(1):398. 10.1186/s12916-025-04200-0. 40691794 10.1186/s12916-025-04200-0 PMC12281790

[4] Angrisani L, Santonicola A, Iovino P, Palma R, Kow L, Prager G, Ramos A, Shikora S, Fiolo F, Harraca JL, et al. IFSO worldwide survey 2020–2021: current trends for bariatric and metabolic procedures. Obes Surg. 2024;34(4):1075–85. 10.1007/s11695-024-07118-3. 38438667 10.1007/s11695-024-07118-3 PMC11026210

[5] Hedbäck N, Dichman ML, Hindsø M, Dirksen C, Jørgensen NB, Bojsen-Møller KN, Kristiansen VB, Rehfeld JF, Hartmann B, Holst JJ, et al. Effect of Ghrelin on glucose tolerance, gut hormones, appetite, and food intake after sleeve gastrectomy. Am J Physiol Endocrinol Metabolism. 2024;327(3):E396–410. 10.1152/ajpendo.00177.2024. 10.1152/ajpendo.00177.2024 PMC11427089 39082900

[6] Casella G, Soricelli E, Giannotti D, Collalti M, Maselli R, Genco A, Redler A, Basso N. Long-term results after laparoscopic sleeve gastrectomy in a large monocentric series. Surg Obes Relat Dis. 2016;12(4):757–62. 10.1016/j.soard.2015.09.028. 26806727 10.1016/j.soard.2015.09.028

[7] Clapp B, Wynn M, Martyn C, Foster C, O’Dell M, Tyroch A. Long term (7 or more years) outcomes of the sleeve gastrectomy: a meta-analysis. Surg Obes Relat Dis. 2018;14(6):741–7. 10.1016/j.soard.2018.02.027. 29625744 10.1016/j.soard.2018.02.027

[8] Peterli R, Wölnerhanssen BK, Peters T, Vetter D, Kröll D, Borbély Y, Schultes B, Beglinger C, Drewe J, Schiesser M, et al. Effect of laparoscopic sleeve gastrectomy vs laparoscopic Roux-en-Y gastric bypass on weight loss in patients with morbid obesity: the SM-BOSS randomized clinical trial. JAMA. 2018;319(3):255–65. 10.1001/jama.2017.20897. 29340679 10.1001/jama.2017.20897 PMC5833546

[9] Catheline JM, Fysekidis M, Dbouk R, Boschetto A, Bihan H, Reach G, Cohen R. Weight loss after sleeve gastrectomy in super superobesity. J Obes. 2012;2012:959260. 10.1155/2012/959260. 22888410 10.1155/2012/959260 PMC3409558

[10] Arapis K, Macrina N, Kadouch D, Ribeiro Parenti L, Marmuse JP, Hansel B. Outcomes of Roux-en-Y gastric bypass versus sleeve gastrectomy in super-super-obese patients (BMI ≥ 60 kg/m(2)): 6-year follow-up at a single university. Surg Obes Relat Dis. 2019;15(1):23–33. 10.1016/j.soard.2018.09.487. 30454974 10.1016/j.soard.2018.09.487

[11] Lemanu DP, Srinivasa S, Singh PP, MacCormick AD, Ulmer S, Morrow J, Hill AG, Babor R, Rahman H. Single-stage laparoscopic sleeve gastrectomy: safety and efficacy in the super-obese. J Surg Res. 2012;177(1):49–54. 10.1016/j.jss.2012.01.011. 22445455 10.1016/j.jss.2012.01.011

[12] Ece I, Yilmaz H, Alptekin H, Yormaz S, Colak B, Yilmaz F, Sahin M. Comparative effectiveness of laparoscopic sleeve gastrectomy on morbidly obese, Super-Obese, and Super-Super obese patients for the treatment of morbid obesity. Obes Surg. 2018;28(6):1484–91. 10.1007/s11695-017-3053-3. 29235011 10.1007/s11695-017-3053-3

[13] Samuel N, Jalal Q, Gupta A, Mazari F, Vasas P, Balachandra S. Mid-term bariatric surgery outcomes for obese patients: does weight matter? Ann R Coll Surg Engl. 2020;102(1):54–61. 10.1308/rcsann.2019.0100. 31891669 10.1308/rcsann.2019.0100 PMC6937615

[14] Khan IA, K AA, Asghar M, Abbas K. Comparative effectiveness of laparoscopic sleeve gastrectomy in morbidly obese and super obese patients. Cureus. 2021;13(12):e20767. 10.7759/cureus.20767. 35111452 10.7759/cureus.20767 PMC8794445

[15] Hoyuela C. Five-year outcomes of laparoscopic sleeve gastrectomy as a primary procedure for morbid obesity: A prospective study. World J Gastrointest Surg. 2017;9(4):109–17. 10.4240/wjgs.v9.i4.109. 28503259 10.4240/wjgs.v9.i4.109 PMC5406732

[16] Athanasiadis DI, Martin A, Kapsampelis P, Monfared S, Stefanidis D. Factors associated with weight regain post-bariatric surgery: a systematic review. Surg Endosc. 2021;35(8):4069–84. 10.1007/s00464-021-08329-w. 33650001 10.1007/s00464-021-08329-w

[17] Yarigholi F, Bahardoust M, Mosavari H, Tehrani FM, Gholizadeh H, Shahmiri SS, Rezvani H, Oshidari B, Garakani K, Eghbali F. Predictors of weight regain and insufficient weight loss according to different definitions after sleeve gastrectomy: a retrospective analytical study. Obes Surg. 2022;32(12):4040–6. 10.1007/s11695-022-06322-3. 36260221 10.1007/s11695-022-06322-3

[18] Chang WW, Hawkins DN, Brockmeyer JR, Faler BJ, Hoppe SW, Prasad BM. Factors influencing long-term weight loss after bariatric surgery. Surg Obes Relat Dis. 2019;15(3):456–61. 10.1016/j.soard.2018.12.033. 30713118 10.1016/j.soard.2018.12.033

[19] Althumiri NA, Bindhim NF, Al-Rayes SA, Alumran A. A systematic review exploring dietary behaviors, psychological determinants and lifestyle factors associated with weight regain after bariatric surgery. Healthcare. 2024;12(22):2243. 39595441 10.3390/healthcare12222243 PMC11594053

[20] Torensma B, Hany M, Berends F, Aarts E, Fink J, Boerma EG. Clarifying terminology in bariatric metabolic surgery: the need for distinction between band and ring. Obes Surg. 2024;34(5):1958–9. 10.1007/s11695-024-07168-7. 38499945 10.1007/s11695-024-07168-7

[21] Fink JM, Hetzenecker A, Seifert G, Runkel M, Laessle C, Fichtner-Feigl S, et al. Banded versus nonbanded sleeve gastrectomy: a randomized controlled trial with 3 years of follow-up. Ann Surg. 2020;272(5):690–5. 10.1097/sla.0000000000004174. 32657920 10.1097/SLA.0000000000004174

[22] Hany M, Berends F, Aarts E, Fink J, Boerma G, Torensma E-J. Technical considerations of ring-augmented laparoscopic sleeve gastrectomy: a step-by-step guide for ring placement by various experts. Obes Surg. 2025;35(2):655–7. 10.1007/s11695-025-07674-2. 39821894 10.1007/s11695-025-07674-2 PMC11836129

[23] Hany M, Ibrahim M, Zidan A, Agayaby ASS, Aboelsoud MR, Gaballah M, et al. Two-year results of the banded versus non-banded re-sleeve gastrectomy as a secondary weight loss procedure after the failure of primary sleeve gastrectomy: a randomized controlled trial. Obes Surg. 2023;33(7):2049–63. 10.1007/s11695-023-06598-z. 37156932 10.1007/s11695-023-06598-z PMC10166688

[24] Fink JM, Reutebuch M, Seifert G, Laessle C, Fichtner-Feigl S, Marjanovic G, Fink M. Banded versus Non-banded sleeve gastrectomy: 5-Year results of a 3-Year randomized controlled trial. Obes Surg. 2024;34(2):310–7. 10.1007/s11695-023-06982-9. 38109013 10.1007/s11695-023-06982-9 PMC10810940

[25] Gawdat K, Osman A, Magdy M, Fadl A. A systematic review of banded vs. Non-Banded sleeve gastrectomy regarding long term results. QJM: Int J Med. 2024;117(Supplement1):hcae070226. 10.1093/qjmed/hcae070.226.

[26] Hany M, Zidan A, Abouelnasr AA, Ibrahim M, Torensma B. Managing the complication of band erosion in banded sleeve gastrectomy: a case report. Obes Surg. 2024;34(3):1052–3. 10.1007/s11695-023-07041-z. 38206565 10.1007/s11695-023-07041-z PMC10899351

[27] Hopewell S, Chan AW, Collins GS, Hróbjartsson A, Moher D, Schulz KF, Tunn R, Aggarwal R, Berkwits M, Berlin JA, et al. CONSORT 2025 statement: updated guideline for reporting randomized trials. JAMA. 2025;333(22):1998–2005. 10.1001/jama.2025.4347. 40228499 10.1001/jama.2025.4347

[28] CONSORT Statement for Randomized Trials of Nonpharmacologic Treatments. A 2017 update and a CONSORT extension for nonpharmacologic trial abstracts. Ann Intern Med. 2017;167(1):40–7. 10.7326/m17-0046/m28630973. 28630973 10.7326/M17-0046

[29] Ware JEJ, Sherbourne CD. The MOS 36-ltem Short-Form health survey (SF-36): I. Conceptual framework and item selection. Med Care. 1992;30(6):473–83. 1593914

[30] AboAbat A, Qannam H, Bjorner JB, Al-Tannir M. Psychometric validation of a Saudi Arabian version of the sf-36v2 health survey and norm data for Saudi Arabia. J patient-reported Outcomes. 2020;4(1):67. 10.1186/s41687-020-00233-6. 10.1186/s41687-020-00233-6 PMC7426352 32789705

[31] Suter M, Calmes JM, Paroz A, Giusti V. A new questionnaire for quick assessment of food tolerance after bariatric surgery. Obes Surg. 2007;17(1):2–8. 10.1007/s11695-007-9016-3. 17355761 10.1007/s11695-007-9016-3

[32] Hany M, Torensma B, Zidan A, Agayby ASS, Ibrahim M, Shafie ME, et al. Comparison of sleeve volume between banded and non-banded sleeve gastrectomy: midterm effect on weight and food tolerance-a retrospective study. Obes Surg. 2023;33(2):406–17. 10.1007/s11695-022-06404-2. 36508154 10.1007/s11695-022-06404-2 PMC9889434

[33] Salminen P, Kow L, Aminian A, Kaplan LM, Nimeri A, Prager G, et al. IFSO consensus on definitions and clinical practice guidelines for obesity management-an international Delphi study. Obes Surg. 2024;34(1):30–42. 10.1007/s11695-023-06913-8. 37999891 10.1007/s11695-023-06913-8 PMC10781804

[34] Lim CH, Lee PC, Lim E, Eng A, Chan WH, Tan HC, Ho E, Kovalik J-P, Ganguly S, Tan J. Resolution of erosive esophagitis after conversion from vertical sleeve gastrectomy to Roux-en-Y gastric bypass. Obes Surg. 2020;30(12):4751–9. 10.1007/s11695-020-04913-6. 32803710 10.1007/s11695-020-04913-6 PMC7429122

[35] Falk V, Twells L, Gregory D, Murphy R, Smith C, Boone D, et al. Laparoscopic sleeve gastrectomy at a new bariatric surgery centre in Canada: 30-day complication rates using the Clavien-Dindo classification. Can J Surg. 2016;59(2):93–7. 10.1503/cjs.016815. 27007089 10.1503/cjs.016815 PMC4814277

[36] Moursi DMA-E, Allam KE, Hetta W, Elsalam AMA, Hussein RS. Role of 3D-CT gastric volumetric study in post-sleeve gastrectomy. Egypt J Radiol Nuclear Med. 2022;53(1):144. 10.1186/s43055-022-00811-2.

[37] Karcz WK, Kuesters S, Marjanovic G, Suesslin D, Kotter E, Thomusch O, Hopt UT, Felmerer G, Langer M, Baumann T. 3D-MSCT gastric pouch volumetry in bariatric surgery-preliminary clinical results. Obes Surg. 2009;19(4):508–16. 10.1007/s11695-008-9776-4. 19104904 10.1007/s11695-008-9776-4

[38] Sabry AA, Emara D. Volumetric pouch study after laparoscopic sleeve gastrectomy. Egypt J Surg. 2018;37(2):265–9. 10.4103/ejs.ejs_29_18.

[39] Lemmens L, Van Den Bossche J, Zaveri H, Surve A. Banded sleeve gastrectomy: better Long-Term results? A Long-Term cohort study until 5 years Follow-Up in obese and superobese patients. Obes Surg. 2018;28(9):2687–95. 10.1007/s11695-018-3248-2. 29671124 10.1007/s11695-018-3248-2 PMC6132784

[40] Chaouch MA, Yang W, Gouader A, Krimi B, Carneiro da Costa A, Pourcher G, Oweira H. Banded versus non-banded sleeve gastrectomy: A systematic review and meta-analysis. Med (Baltim). 2023;102(15):e32982. 10.1097/md.0000000000032982. 10.1097/MD.0000000000032982 PMC10101294 37058050

[41] Noria SF, Shelby RD, Atkins KD, Nguyen NT, Gadde KM. Weight regain after bariatric surgery: scope of the problem, causes, prevention, and treatment. Curr Diab Rep. 2023;23(3):31–42. 10.1007/s11892-023-01498-z. 36752995 10.1007/s11892-023-01498-z PMC9906605

[42] Hany M, Sabry A, Torensma B, Ahmed K, Refaie M, Zidan A, Agayby ASS, Ibrahim M, Mourad M. Comparison of the mid-term outcomes of banded and non-banded sleeve gastrectomy: safety, food tolerance, and weight regain. Surg Endosc. 2022;36(12):9146–55. 10.1007/s00464-022-09395-4. 35764843 10.1007/s00464-022-09395-4 PMC9652222

[43] Dargent J. Isolated food intolerance after adjustable gastric banding: a major cause of long-term band removal. Obes Surg. 2008;18(7):829–32. 10.1007/s11695-008-9495-x. 18459020 10.1007/s11695-008-9495-x

[44] Stroh C, Hohmann U, Schramm H, Meyer F, Manger T. Fourteen-year long-term results after gastric banding. J Obes. 2011;2011:128451. 10.1155/2011/128451. 21234392 10.1155/2011/128451 PMC3017910

[45] Martikainen T, Pirinen E, Alhava E, Poikolainen E, Pääkkönen M, Uusitupa M, Gylling H. Long-term results, late complications and quality of life in a series of adjustable gastric banding. Obes Surg. 2004;14(5):648–54. 10.1381/096089204323093435. 15186633 10.1381/096089204323093435

[46] Simadibrata DM, Lesmana E, Fass R. Role of endoscopy in gastroesophageal reflux disease. Clin Endosc. 2023;56(6):681–92. 10.5946/ce.2023.182. 37822063 10.5946/ce.2023.182 PMC10665616

[47] Chen JW, Vela MF, Peterson KA, Carlson DA. AGA clinical practice update on the diagnosis and management of extraesophageal gastroesophageal reflux disease: expert review. Clin Gastroenterol Hepatol. 2023;21(6):1414–e14211413. 10.1016/j.cgh.2023.01.040. 37061897 10.1016/j.cgh.2023.01.040

[48] Gyawali CP, Yadlapati R, Fass R, Katzka D, Pandolfino J, Savarino E. Updates to the modern diagnosis of GERD: Lyon consensus 2.0. Gut. 2024;73:361–71. 37734911 10.1136/gutjnl-2023-330616 PMC10846564

[49] Dalal JJ, Padmanabhan TN, Jain P, Patil S, Vasnawala H, Gulati A. LIPITENSION: interplay between dyslipidemia and hypertension. Indian J Endocrinol Metab. 2012;16(2):240–5. 10.4103/2230-8210.93742. 22470861 10.4103/2230-8210.93742 PMC3313742

[50] Otsuka T, Takada H, Nishiyama Y, Kodani E, Saiki Y, Kato K, Kawada T. Dyslipidemia and the risk of developing hypertension in a Working-Age male population. J Am Heart Assoc. 2016;5(3):e003053. 10.1161/jaha.115.003053. 27016576 10.1161/JAHA.115.003053 PMC4943276

[51] Dąbrowska E, Narkiewicz K. Hypertension and dyslipidemia: the two partners in endothelium-related crime. Curr Atheroscler Rep. 2023;25(9):605–12. 10.1007/s11883-023-01132-z. 37594602 10.1007/s11883-023-01132-z PMC10471742

[52] Shariq OA, McKenzie TJ. Obesity-related hypertension: a review of pathophysiology, management, and the role of metabolic surgery. Gland Surg. 2020;9(1):80–93. 10.21037/gs.2019.12.03. 32206601 10.21037/gs.2019.12.03 PMC7082272

[53] Vekic J, Zeljkovic A, Stefanovic A, Jelic-Ivanovic Z, Spasojevic-Kalimanovska V. Obesity and dyslipidemia. Metabolism. 2019;92:71–81. 10.1016/j.metabol.2018.11.005. 30447223 10.1016/j.metabol.2018.11.005

[54] Felsenreich DM, Prager G, Kefurt R, Eilenberg M, Jedamzik J, Beckerhinn P, et al. Quality of life 10 years after sleeve gastrectomy: a multicenter study. Obes Facts. 2019;12(2):157–66. 10.1159/000496296. 30879011 10.1159/000496296 PMC6547272

[55] AboKhozima A, Zidan MH, Altabbaa H, Selim A, Alokl M, Mourad M, Elmagd AA, Elsayed MEG, Emara AF, Eskander GM, et al. The impact of weight loss after bariatric surgeries on the patient’s body image, quality of life, and self-esteem. Langenbeck’s Archives Surg. 2025;410(1):24. 10.1007/s00423-024-03568-6. 10.1007/s00423-024-03568-6 PMC11700042 39755894

[56] Kirkil C, Aygen E, Korkmaz MF, Bozan MB. Quality of life after laparoscopic sleeve gastrectomy using baros system. Arquivos Brasileiros De Cirurgia Digestiva: ABCD = Brazilian Archives Dig Surg. 2018;31(3):e1385. 10.1590/0102-672020180001e1385. 10.1590/0102-672020180001e1385 PMC6097161 30133677

