# Supplementary material for: Ring-Augmented Versus Non-Ring Augmented Sleeve Gastrectomy in Patients with BMI > 50 kg/m²: 3-Year Follow-up of a Randomized Controlled Trial
Source: Obes Surg. 2026 Jan 20;36(3):1035–53. doi: 10.1007/s11695-025-08431-1 (PMC13038747; doi:10.1007/s11695-025-08431-1)
Supplement: Supplementary file 1 — Supplementary Material 1 [file 11695_2025_8431_MOESM1_ESM.docx]

# Supplementary File -1

| **Table S1:** Weight loss outcomes estimates from GEE analyses for the within and between the SG and Ra-SG groups (complete data analysis). Cell values represent Mean ± standard deviations. *Statistically significant (p < 0.05). MD: Mean difference. CI: Confidence Interval | | | | | | | | | |
| --- | --- | --- | --- | --- | --- | --- | --- | --- | --- |
| **Variable** | **Time** | **SG** | | | **Ra-SG** | | | **MD between SG and Ra-SG**  **(95% CI)** | **p** |
|  |  | **M ± SD** | **MD from baseline** | **p** | **M ± SD** | **MD from baseline** | **p** |  |  |
| **Weight (Kg)** | Baseline | 154.3 ± 19.4 | Reference |  | 154.7 ± 19.5 | Reference |  | -0.43 (-5.56, 4.71) | 0.870 |
|  | Six months | 112.5 ± 11.5 | -41.79 (-47.10, -36.48) | < 0.001* | 113.4 ± 12.2 | -41.26 (-46.60, -35.92) | < 0.001* | -0.96 (-4.09, 2.18) | 0.550 |
|  | Year 1 | 78.2 ± 8.8 | -76.07 (-81.09, -71.05) | < 0.001* | 78.5 ± 10.9 | -76.21 (-81.41, -71.00) | < 0.001* | -0.29 (-2.94, 2.36) | 0.830 |
|  | Year 2 | 76.4 ± 8.8 | -77.88 (-82.93, -72.83) | < 0.001* | 76.0 ± 9.2 | -78.65 (-83.70, -73.61) | < 0.001* | 0.34 (-2.14, 2.83) | 0.786 |
|  | Year 3 | 83.3 ± 12.9 | -70.98 (-76.55, -65.42) | < 0.001* | 77.9 ± 11.3 | -76.82 (-82.10, -71.53) | < 0.001* | 5.40 (2.05, 8.76) | 0.002* |
| **BMI (kg/m²)** | Baseline | 53.7 ± 2.8 | Reference |  | 53.8 ± 2.9 | Reference |  | -0.07 (-0.83, 0.68) | 0.846 |
|  | Six months | 39.4 ± 3.6 | -14.35 (-15.42, -13.27) | < 0.001* | 39.7 ± 3.7 | -14.11 (-15.21, -13.01) | < 0.001* | -0.31 (-1.28, 0.65) | 0.524 |
|  | Year 1 | 27.4 ± 3.3 | -26.31 (-27.35, -25.28) | < 0.001* | 27.6 ± 3.9 | -26.21 (-27.35, -25.07) | < 0.001* | -0.18 (-1.15, 0.79) | 0.716 |
|  | Year 2 | 26.8 ± 3.2 | -26.95 (-27.98, -25.91) | < 0.001* | 26.9 ± 3.9 | -26.94 (-28.11, -25.77) | < 0.001* | -0.08 (-1.08, 0.91) | 0.873 |
|  | Year 3 | 29.3 ± 4.7 | -24.47 (-25.80, -23.13) | < 0.001* | 27.5 ± 4.4 | -26.31 (-27.57, -25.06) | < 0.001* | 1.77 (0.52, 3.03) | 0.006* |
| **TWL(%)** | Six months | 26.6 ± 7.1 | Reference |  | 26.7 ± 9.2 | Reference |  | -0.18 (-2.35, 1.98) | 0.868 |
|  | Year 1 | 48.8 ± 6.9 | 22.29 (20.04, 24.54) | < 0.001* | 48.5 ± 7.6 | 21.78 (19.09, 24.46) | < 0.001* | 0.33 (-1.62, 2.27) | 0.742 |
|  | Year 2 | 50.1 ± 6.6 | 23.52 (21.28, 25.76) | < 0.001* | 49.9 ± 7.7 | 23.18 (20.45, 25.91) | < 0.001* | 0.16 (-1.82, 2.14) | 0.875 |
|  | Year 3 | 45.5 ± 9.0 | 18.99 (16.32, 21.65) | < 0.001* | 48.8 ± 8.3 | 22.05 (19.23, 24.86) | < 0.001* | -3.25 (-5.63, -0.86) | 0.008* |

| **Table S2: Weight loss outcomes estimates from GEE analyses for the within and between the SG and Ra-SG groups (complete cases analysis)** | | | | | | | | | |
| --- | --- | --- | --- | --- | --- | --- | --- | --- | --- |
| **Variable** | **Time** | **SG** | | | **Ra-SG** | | | **MD between SG and Ra-SG**  **(95% CI)** | **p** |
|  |  | **M ± SD** | **MD from baseline** | **p** | **M ± SD** | **MD from baseline** | **p** |  |  |
| **Weight (Kg)** | Baseline | 154.5 ± 19.3 | Reference |  | 153.7 ± 19.3 | Reference |  | 0.73 (-4.59, 6.06) | 0.787 |
|  | Six months | 112.5 ± 11.7 | -42.01 (-47.58, -36.44) | < 0.001* | 112.7 ± 11.3 | -41.02 (-46.44, -35.60) | < 0.001* | -0.25 (-3.43, 2.93) | 0.878 |
|  | Year 1 | 77.9 ± 8.8 | -76.63 (-81.86, -71.39) | < 0.001* | 78.2 ± 11.0 | -75.52 (-80.90, -70.15) | < 0.001* | -0.37 (-3.11, 2.38) | 0.793 |
|  | Year 2 | 76.4 ± 8.8 | -78.08 (-83.31, -72.86) | < 0.001* | 76.0 ± 9.2 | -77.69 (-82.87, -72.51) | < 0.001* | 0.34 (-2.14, 2.83) | 0.786 |
|  | Year 3 | 83.3 ± 12.9 | -71.19 (-76.92, -65.46) | < 0.001* | 77.9 ± 11.3 | -75.86 (-81.27, -70.44) | < 0.001* | 5.40 (2.05, 8.76) | 0.002* |
| **BMI (kg/m²)** | Baseline | 53.8 ± 2.8 | Reference |  | 53.8 ± 2.9 | Reference |  | 0.01 (-0.78, 0.81) | 0.980 |
|  | Six months | 39.4 ± 3.5 | -14.44 (-15.56, -13.32) | < 0.001* | 39.7 ± 3.7 | -14.08 (-15.22, -12.94) | < 0.001* | -0.35 (-1.35, 0.65) | 0.495 |
|  | Year 1 | 27.3 ± 3.3 | -26.50 (-27.57, -25.43) | < 0.001* | 27.6 ± 3.9 | -26.23 (-27.41, -25.05) | < 0.001* | -0.26 (-1.26, 0.73) | 0.607 |
|  | Year 2 | 26.8 ± 3.2 | -27.01 (-28.08, -25.94) | < 0.001* | 26.9 ± 3.9 | -26.92 (-28.10, -25.73) | < 0.001* | -0.08 (-1.08, 0.91) | 0.873 |
|  | Year 3 | 29.3 ± 4.7 | -24.53 (-25.89, -23.17) | < 0.001* | 27.5 ± 4.4 | -26.29 (-27.56, -25.02) | < 0.001* | 1.77 (0.52, 3.03) | 0.006* |
| **TWL(%)** | Six months | 26.7 ± 6.7 | Reference |  | 26.8 ± 9.5 | Reference |  | -0.04 (-2.29, 2.22) | 0.975 |
|  | Year 1 | 49.1 ± 6.6 | 22.41 (20.17, 24.65) | < 0.001* | 48.6 ± 7.7 | 21.89 (19.04, 24.74) | < 0.001* | 0.48 (-1.50, 2.47) | 0.632 |
|  | Year 2 | 50.1 ± 6.6 | 23.36 (21.13, 25.60) | < 0.001* | 49.9 ± 7.7 | 23.17 (20.33, 26.01) | < 0.001* | 0.16 (-1.82, 2.14) | 0.875 |
|  | Year 3 | 45.5 ± 9.0 | 18.83 (16.17, 21.49) | < 0.001* | 48.8 ± 8.3 | 22.04 (19.11, 24.96) | < 0.001* | -3.25 (-5.63, -0.86) | 0.008* |

Cell values represent Mean ± standard deviations. *Statistically significant (p < 0.05). MD: Mean difference. CI: Confidence Interval

| **Table S3: Sf36 domains’ estimates from GEE analyses for the within and between the SG and Ra-SG groups (Complete data analysis)** | | | | | | | | | |
| --- | --- | --- | --- | --- | --- | --- | --- | --- | --- |
| **Variable** | **Time** | **SG** | | | **Ra-SG** | | | **MD between SG and Ra-SG**  **(95% CI)** | **p** |
|  |  | **M ± SD** | **MD from baseline** | **p** | **M ± SD** | **MD from baseline** | **p** |  |  |
| **Physical Functioning** | Baseline | 41.3 ± 11.8 | Reference |  | 41.0 ± 12.5 | Reference |  | 0.35 (-2.87, 3.56) | 0.832 |
|  | Year 1 | 65.9 ± 7.6 | 24.61 (21.60, 27.62) | < 0.001* | 65.4 ± 8.0 | 24.41 (21.27, 27.55) | < 0.001* | 0.54 (-1.55, 2.63) | 0.609 |
|  | Year 3 | 76.6 ± 8.0 | 35.33 (32.24, 38.42) | < 0.001* | 74.9 ± 9.3 | 33.95 (30.63, 37.28) | < 0.001* | 1.72 (-0.66, 4.11) | 0.157 |
| **Role Physical** | Baseline | 39.9 ± 10.3 | Reference |  | 40.7 ± 11.2 | Reference |  | -0.82 (-3.67, 2.03) | 0.572 |
|  | Year 1 | 64.6 ± 8.4 | 24.65 (21.78, 27.51) | < 0.001* | 63.6 ± 8.9 | 22.82 (19.79, 25.85) | < 0.001* | 1.01 (-1.32, 3.33) | 0.395 |
|  | Year 3 | 73.8 ± 8.4 | 33.91 (31.01, 36.80) | < 0.001* | 73.4 ± 8.6 | 32.62 (29.60, 35.64) | < 0.001* | 0.46 (-1.88, 2.81) | 0.698 |
| **Pain** | Baseline | 36.6 ± 11.5 | Reference |  | 40.2 ± 11.8 | Reference |  | **-3.58 (-6.66, -0.50)** | **0.023*** |
|  | Year 1 | 65.8 ± 8.9 | 29.21 (26.10, 32.32) | < 0.001* | 65.7 ± 8.9 | 25.52 (22.38, 28.65) | < 0.001* | 0.11 (-2.27, 2.49) | 0.926 |
|  | Year 3 | 76.4 ± 8.7 | 39.81 (36.67, 42.94) | < 0.001* | 74.9 ± 9.1 | 34.69 (31.50, 37.88) | < 0.001* | 1.54 (-0.93, 4.00) | 0.221 |
| **General Health** | Baseline | 40.8 ± 11.0 | Reference |  | 40.9 ± 11.2 | Reference |  | -0.10 (-3.04, 2.84) | 0.946 |
|  | Year 1 | 66.2 ± 8.5 | 25.40 (22.42, 28.39) | < 0.001* | 64.9 ± 8.3 | 24.01 (21.06, 26.97) | < 0.001* | 1.29 (-0.96, 3.54) | 0.262 |
|  | Year 3 | 74.4 ± 10.9 | 33.64 (30.26, 37.02) | < 0.001* | 73.6 ± 9.8 | 32.75 (29.56, 35.95) | < 0.001* | 0.78 (-2.08, 3.64) | 0.591 |
| **Social Functioning** | Baseline | 39.8 ± 12.0 | Reference |  | 39.1 ± 12.2 | Reference |  | 0.75 (-2.46, 3.95) | 0.647 |
|  | Year 1 | 64.5 ± 8.3 | 24.70 (21.58, 27.83) | < 0.001* | 63.3 ± 9.4 | 24.24 (20.98, 27.50) | < 0.001* | 1.21 (-1.15, 3.58) | 0.314 |
|  | Year 3 | 75.3 ± 9.6 | 35.48 (32.13, 38.82) | < 0.001* | 75.4 ± 10.0 | 36.32 (32.94, 39.71) | < 0.001* | -0.10 (-2.81, 2.61) | 0.944 |
| **Role Emotional** | Baseline | 38.8 ± 12.3 | Reference |  | 41.6 ± 11.3 | Reference |  | -2.79 (-5.91, 0.34) | 0.080 |
|  | Year 1 | 67.0 ± 9.1 | 28.26 (24.98, 31.53) | < 0.001* | 64.8 ± 9.2 | 23.20 (20.12, 26.29) | < 0.001* | 2.27 (-0.17, 4.71) | 0.069 |
|  | Year 3 | 73.9 ± 10.0 | 35.13 (31.68, 38.57) | < 0.001* | 73.1 ± 9.6 | 31.54 (28.37, 34.72) | < 0.001* | 0.80 (-1.91, 3.50) | 0.562 |
| **Vitality** | Baseline | 39.3 ± 10.8 | Reference |  | 38.8 ± 11.4 | Reference |  | 0.55 (-2.38, 3.48) | 0.713 |
|  | Year 1 | 65.2 ± 7.8 | 25.87 (23.03, 28.72) | < 0.001* | 66.2 ± 8.5 | 27.40 (24.39, 30.42) | < 0.001* | -0.98 (-3.17, 1.21) | 0.379 |
|  | Year 3 | 74.7 ± 8.4 | 35.37 (32.40, 38.34) | < 0.001* | 75.9 ± 8.1 | 37.08 (34.09, 40.07) | < 0.001* | -1.16 (-3.45, 1.13) | 0.320 |
| **Mental Health** | Baseline | 39.9 ± 11.3 | Reference |  | 40.7 ± 10.9 | Reference |  | -0.80 (-3.72, 2.13) | 0.593 |
|  | Year 1 | 66.3 ± 8.6 | 26.42 (23.39, 29.46) | < 0.001* | 66.1 ± 8.9 | 25.41 (22.43, 28.38) | < 0.001* | 0.22 (-2.13, 2.56) | 0.857 |
|  | Year 3 | 76.2 ± 9.2 | 36.28 (33.11, 39.44) | < 0.001* | 76.4 ± 8.5 | 35.65 (32.70, 38.60) | < 0.001* | -0.17 (-2.61, 2.27) | 0.891 |
| **PCS** | Baseline | 42.6 ± 12.5 | Reference |  | 44.7 ± 12.1 | Reference |  | -2.09 (-5.35, 1.16) | 0.207 |
|  | Year 1 | 66.8 ± 8.9 | 24.25 (20.96, 27.55) | < 0.001* | 65.6 ± 8.3 | 20.93 (17.83, 24.04) | < 0.001* | 1.23 (-1.08, 3.53) | 0.296 |
|  | Year 3 | 72.4 ± 13.4 | 29.82 (25.80, 33.83) | < 0.001* | 72.9 ± 11.1 | 28.24 (24.71, 31.78) | < 0.001* | -0.52 (-3.93, 2.89) | 0.766 |
| **MCS** | Baseline | 42.3 ± 13.5 | Reference |  | 40.1 ± 12.9 | Reference |  | 2.15 (-1.33, 5.63) | 0.226 |
|  | Year 1 | 67.0 ± 9.1 | 24.75 (21.27, 28.24) | < 0.001* | 67.5 ± 8.4 | 27.42 (24.17, 30.67) | < 0.001* | -0.52 (-2.87, 1.83) | 0.663 |
|  | Year 3 | 75.4 ± 8.5 | 33.18 (29.74, 36.63) | < 0.001* | 72.9 ± 12.8 | 32.82 (28.91, 36.72) | < 0.001* | 2.52 (-0.48, 5.51) | 0.099 |

Cell values represent Mean ± standard deviations. *Statistically significant (p < 0.05). MD: Mean difference. CI: Confidence Interval

| **Table S4: Sf36 domains’ estimates from GEE analyses for the within and between the SG and Ra-SG groups (Complete cases analysis)** | | | | | | | | | |
| --- | --- | --- | --- | --- | --- | --- | --- | --- | --- |
| **Variable** | **Time** | **SG** | | | **Ra-SG** | | | **MD between SG and Ra-SG**  **(95% CI)** | **p** |
|  |  | **M ± SD** | **MD from baseline** | **p** | **M ± SD** | **MD from baseline** | **p** |  |  |
| **Physical Functioning** | Baseline | 40.9 ± 12.1 | Reference |  | 41.0 ± 12.2 | Reference |  | 0.00 (-3.37, 3.36) | 0.998 |
|  | Year 1 | 65.9 ± 7.4 | 24.94 (21.76, 28.12) | < 0.001* | 65.6 ± 7.9 | 24.64 (21.44, 27.84) | < 0.001* | 0.30 (-1.83, 2.43) | 0.783 |
|  | Year 3 | 76.6 ± 8.0 | 35.68 (32.43, 38.93) | < 0.001* | 74.9 ± 9.3 | 33.96 (30.59, 37.32) | < 0.001* | 1.72 (-0.66, 4.11) | 0.157 |
| **Role Physical** | Baseline | 40.0 ± 10.4 | Reference |  | 40.8 ± 11.0 | Reference |  | -0.75 (-3.71, 2.20) | 0.617 |
|  | Year 1 | 64.3 ± 8.4 | 24.31 (21.30, 27.31) | < 0.001* | 63.5 ± 9.0 | 22.78 (19.67, 25.89) | < 0.001* | 0.77 (-1.63, 3.18) | 0.528 |
|  | Year 3 | 73.8 ± 8.4 | 33.82 (30.83, 36.82) | < 0.001* | 73.4 ± 8.6 | 32.60 (29.55, 35.66) | < 0.001* | 0.46 (-1.88, 2.81) | 0.698 |
| **Pain** | Baseline | 36.8 ± 11.2 | Reference |  | 40.6 ± 12.0 | Reference |  | **-3.82 (-7.03, -0.60)** | **0.020*** |
|  | Year 1 | 65.9 ± 8.7 | 29.19 (26.01, 32.38) | < 0.001* | 65.8 ± 9.0 | 25.24 (21.95, 28.53) | < 0.001* | 0.14 (-2.31, 2.58) | 0.913 |
|  | Year 3 | 76.4 ± 8.7 | 39.66 (36.47, 42.85) | < 0.001* | 74.9 ± 9.1 | 34.31 (31.00, 37.61) | < 0.001* | 1.54 (-0.93, 4.00) | 0.221 |
| **General Health** | Baseline | 40.8 ± 11.0 | Reference |  | 40.5 ± 11.2 | Reference |  | 0.27 (-2.78, 3.33) | 0.861 |
|  | Year 1 | 66.1 ± 8.5 | 25.29 (22.19, 28.40) | < 0.001* | 64.9 ± 8.3 | 24.46 (21.41, 27.51) | < 0.001* | 1.11 (-1.21, 3.43) | 0.347 |
|  | Year 3 | 74.4 ± 10.9 | 33.63 (30.18, 37.09) | < 0.001* | 73.6 ± 9.8 | 33.12 (29.87, 36.38) | < 0.001* | 0.78 (-2.08, 3.64) | 0.591 |
| **Social Functioning** | Baseline | 39.5 ± 12.2 | Reference |  | 39.0 ± 12.3 | Reference |  | 0.51 (-2.88, 3.90) | 0.769 |
|  | Year 1 | 64.5 ± 8.3 | 25.06 (21.76, 28.37) | < 0.001* | 63.8 ± 9.3 | 24.85 (21.46, 28.24) | < 0.001* | 0.72 (-1.72, 3.16) | 0.565 |
|  | Year 3 | 75.3 ± 9.6 | 35.84 (32.37, 39.32) | < 0.001* | 75.4 ± 10.0 | 36.45 (32.97, 39.93) | < 0.001* | -0.10 (-2.81, 2.61) | 0.944 |
| **Role Emotional** | Baseline | 39.6 ± 12.3 | Reference |  | 41.8 ± 11.3 | Reference |  | -2.18 (-5.45, 1.09) | 0.192 |
|  | Year 1 | 66.8 ± 8.9 | 27.19 (23.78, 30.59) | < 0.001* | 64.7 ± 9.1 | 22.85 (19.66, 26.04) | < 0.001* | 2.15 (-0.34, 4.65) | 0.090 |
|  | Year 3 | 73.9 ± 10.0 | 34.28 (30.73, 37.83) | < 0.001* | 73.1 ± 9.6 | 31.30 (28.05, 34.55) | < 0.001* | 0.80 (-1.91, 3.50) | 0.562 |
| **Vitality** | Baseline | 39.6 ± 10.8 | Reference |  | 38.8 ± 11.7 | Reference |  | 0.86 (-2.25, 3.97) | 0.587 |
|  | Year 1 | 65.3 ± 7.9 | 25.64 (22.64, 28.64) | < 0.001* | 66.3 ± 8.5 | 27.52 (24.33, 30.70) | < 0.001* | -1.01 (-3.30, 1.27) | 0.383 |
|  | Year 3 | 74.7 ± 8.4 | 35.08 (32.01, 38.15) | < 0.001* | 75.9 ± 8.1 | 37.10 (33.98, 40.22) | < 0.001* | -1.16 (-3.45, 1.13) | 0.320 |
| **Mental Health** | Baseline | 40.0 ± 11.2 | Reference |  | 40.6 ± 10.8 | Reference |  | -0.61 (-3.66, 2.44) | 0.694 |
|  | Year 1 | 66.4 ± 8.6 | 26.38 (23.23, 29.54) | < 0.001* | 66.0 ± 8.9 | 25.39 (22.32, 28.46) | < 0.001* | 0.38 (-2.03, 2.79) | 0.755 |
|  | Year 3 | 76.2 ± 9.2 | 36.16 (32.92, 39.40) | < 0.001* | 76.4 ± 8.5 | 35.72 (32.71, 38.74) | < 0.001* | -0.17 (-2.61, 2.27) | 0.891 |
| **PCS** | Baseline | 43.0 ± 12.4 | Reference |  | 45.1 ± 12.2 | Reference |  | -2.07 (-5.47, 1.33) | 0.233 |
|  | Year 1 | 66.3 ± 8.9 | 23.32 (19.90, 26.73) | < 0.001* | 65.9 ± 8.4 | 20.77 (17.51, 24.02) | < 0.001* | 0.48 (-1.91, 2.88) | 0.691 |
|  | Year 3 | 72.4 ± 13.4 | 29.37 (25.29, 33.45) | < 0.001* | 72.9 ± 11.1 | 27.82 (24.19, 31.46) | < 0.001* | -0.52 (-3.93, 2.89) | 0.766 |
| **MCS** | Baseline | 42.2 ± 13.9 | Reference |  | 40.2 ± 13.1 | Reference |  | 2.05 (-1.68, 5.79) | 0.281 |
|  | Year 1 | 67.2 ± 9.0 | 25.00 (21.29, 28.71) | < 0.001* | 67.6 ± 8.4 | 27.41 (24.00, 30.82) | < 0.001* | -0.35 (-2.76, 2.05) | 0.772 |
|  | Year 3 | 75.4 ± 8.5 | 33.22 (29.57, 36.88) | < 0.001* | 72.9 ± 12.8 | 32.76 (28.75, 36.78) | < 0.001* | 2.52 (-0.48, 5.51) | 0.099 |

Cell values represent Mean ± standard deviations. *Statistically significant (p < 0.05). MD: Mean difference. CI: Confidence Interval

| **Table S5: Food tolerance estimates from GEE analyses for the within and between the SG and Ra-SG groups (Complete data analysis)** | | | | | | | | | |
| --- | --- | --- | --- | --- | --- | --- | --- | --- | --- |
| **Variable** | **Time** | **SG** | | | **Ra-SG** | | | **MD between SG and Ra-SG**  **(95% CI)** | **p** |
|  |  | **M ± SD** | **MD from baseline** | **p** | **M ± SD** | **MD from baseline** | **p** |  |  |
| **Food tolerance** | Year 1 | 21.5 ± 0.5 | Reference |  | 21.0 ± 0.6 | Reference |  | 0.46 (0.31, 0.60) | **< 0.001*** |
|  | Year 2 | 23.3 ± 0.7 | 1.81 (1.62, 2.00) | < 0.001* | 21.6 ± 0.7 | 0.60 (0.41, 0.79) | < 0.001* | 1.67 (1.48, 1.86) | **< 0.001*** |
|  | Year 3 | 24.2 ± 0.9 | 2.70 (2.46, 2.94) | < 0.001* | 22.0 ± 0.6 | 1.03 (0.84, 1.22) | < 0.001* | 2.12 (1.90, 2.35) | **< 0.001*** |

Cell values represent Mean ± standard deviations or frequency (%). *Statistically significant (p < 0.05). MD: Mean difference. CI: Confidence Interval

| **Table S6: Food tolerance estimates from GEE analyses for the within and between the SG and Ra-SG groups (Complete cases analysis)** | | | | | | | | | |
| --- | --- | --- | --- | --- | --- | --- | --- | --- | --- |
| **Variable** | **Time** | **SG** | | | **Ra-SG** | | | **MD between SG and Ra-SG**  **(95% CI)** | **p** |
|  |  | **M ± SD** | **MD from baseline** | **p** | **M ± SD** | **MD from baseline** | **p** |  |  |
| **Food tolerance** | Year 1 | 21.5 ± 0.5 | Reference |  | 21.0 ± 0.6 | Reference |  | 0.48 (0.33, 0.63) | **< 0.001*** |
|  | Year 2 | 23.3 ± 0.7 | 1.81 (1.62, 2.00) | < 0.001* | 21.6 ± 0.7 | 0.62 (0.42, 0.81) | < 0.001* | 1.67 (1.48, 1.86) | **< 0.001*** |
|  | Year 3 | 24.2 ± 0.9 | 2.69 (2.45, 2.93) | < 0.001* | 22.0 ± 0.6 | 1.05 (0.86, 1.24) | < 0.001* | 2.12 (1.90, 2.35) | **< 0.001*** |

Cell values represent Mean ± standard deviations. *Statistically significant (p < 0.05). MD: Mean difference. CI: Confidence Interval

| **Table S7: Comparison between SG and Ra-SG groups in terms of the fate of associated medical problems along comparisons with baseline within groups using McNemar test (complete cases analysis)** | | | | |
| --- | --- | --- | --- | --- |
| **Associated Medical Problem** | **Time** | **SG** | **Ra-SG** | **p** |
| **Cardiac Conditions** | Baseline | 10 (10.2) | 3 (2.9) | 0.046* |
|  | Year1 | 0 (0.0)** | 0 (0.0) |  |
|  | Year3 | 3 (3.1) ** | 2 (2.0) | 0.678 |
| **Diabetes** | Baseline | 12 (12.2) | 24 (23.5) | 0.058 |
|  | Year1 | 2 (2.0) ** | 2 (2.0) ** | 1.000 |
|  | Year3 | 3 (3.1) ** | 3 (2.9) ** | 1.000 |
| **Dyslipidemia** | Baseline | 28 (28.6) | 33 (32.4) | 0.669 |
|  | Year1 | 1 (1.0) ** | 0 (0.0) ** | 0.490 |
|  | Year3 | 8 (8.2) ** | 0 (0.0) ** | 0.003* |
| **Hypertension** | Baseline | 30 (30.6) | 32 (31.4) | 1.000 |
|  | Year1 | 1 (1.0) ** | 3 (2.9) ** | 0.622 |
|  | Year3 | 8 (8.2) ** | 0 (0.0) ** | 0.003* |
| **Osteoarthritis** | Baseline | 29 (29.6) | 22 (21.6) | 0.255 |
|  | Year1 | 5 (5.1) ** | 6 (5.9) ** | 1.000 |
|  | Year3 | 9 (9.2) ** | 4 (3.9) ** | 0.158 |
| **Sleep apnea** | Baseline | 7 (7.1) | 10 (9.8) | 0.674 |
|  | Year1 | 1 (1.0) ** | 2 (2.0) ** | 1.000 |
|  | Year3 | 3 (3.1) | 2 (2.0) ** | 0.678 |

Cell values represent frequency (%). *Statistically significant Chi-square or Fisher’s exact test for the between-groups comparisons at each time point (p < 0.05). **Statistically significant McNemar test for the within-group comparisons vs baseline (p < 0.05)

| **Table S8: Lab results’ estimates from GEE analyses for the within and between the SG and Ra-SG groups (Complete data analysis)** | | | | | | | | | |
| --- | --- | --- | --- | --- | --- | --- | --- | --- | --- |
| **Variable** | **Time** | **SG** | | | **Ra-SG** | | | **MD between SG and Ra-SG(95% CI)** | **p** |
|  |  | **M ± SD** | **MD from baseline** | **p** | **M ± SD** | **MD from baseline** | **p** |  |  |
| **Albumin** | Baseline | 4.5 ± 0.6 | Reference |  | 4.4 ± 0.5 | Reference |  | 0.10 (-0.05, 0.25) | 0.206 |
|  | Six months | 4.4 ± 0.5 | -0.16 (-0.34, 0.03) | 0.128 | 4.5 ± 0.6 | 0.09 (-0.10, 0.28) | 0.580 | -0.15 (-0.30, 0.00) | 0.049* |
|  | Year 1 | 4.2 ± 0.5 | -0.36 (-0.53, -0.18) | < 0.001* | 4.3 ± 0.5 | -0.17 (-0.34, 0.00) | 0.047* | -0.09 (-0.21, 0.04) | 0.163 |
|  | Year 2 | 4.3 ± 0.4 | -0.28 (-0.45, -0.11) | < 0.001* | 4.4 ± 0.5 | 0.00 (-0.17, 0.17) | 1.000 | -0.18 (-0.29, -0.06) | 0.003* |
|  | Year 3 | 4.0 ± 0.4 | -0.50 (-0.68, -0.33) | < 0.001* | 4.0 ± 0.4 | -0.41 (-0.57, -0.26) | < 0.001* | 0.01 (-0.10, 0.12) | 0.883 |
| **VITAMIN B12** | Baseline | 604.9 ± 367.3 | Reference |  | 446.2 ± 154.3 | Reference |  | 158.67 (83.94, 233.40) | < 0.001* |
|  | Six months | 577.1 ± 399.6 | -27.73 (-155.47, 100.00) | 0.920 | 429.3 ± 171.1 | -16.92 (-70.41, 36.57) | 0.813 | 147.85 (66.31, 229.39) | < 0.001* |
|  | Year 1 | 551.1 ± 331.7 | -53.79 (-171.00, 63.41) | 0.602 | 529.5 ± 344.6 | 83.33 (-5.32, 171.99) | 0.073 | 21.54 (-68.97, 112.05) | 0.641 |
|  | Year 2 | 654.4 ± 228.7 | 49.55 (-53.70, 152.80) | 0.569 | 555.0 ± 219.3 | 108.80 (44.76, 172.85) | < 0.001* | 99.41 (37.51, 161.31) | 0.002* |
|  | Year 3 | 591.5 ± 159.1 | -13.40 (-108.35, 81.54) | 0.971 | 543.8 ± 163.1 | 97.54 (44.23, 150.85) | < 0.001* | 47.72 (3.25, 92.20) | 0.035* |
| **Vit D** | Baseline | 31.1 ± 11.7 | Reference |  | 24.6 ± 11.5 | Reference |  | 6.50 (3.43, 9.56) | < 0.001* |
|  | Six months | 25.0 ± 10.9 | -6.07 (-9.83, -2.30) | < 0.001* | 34.2 ± 11.3 | 9.64 (5.89, 13.39) | < 0.001* | -9.21 (-12.14, -6.27) | < 0.001* |
|  | Year 1 | 26.7 ± 10.0 | -4.37 (-8.01, -0.73) | 0.012* | 31.7 ± 15.2 | 7.09 (2.63, 11.56) | < 0.001* | -4.97 (-8.40, -1.54) | 0.005* |
|  | Year 2 | 36.9 ± 8.6 | 5.85 (2.37, 9.32) | < 0.001* | 35.2 ± 10.5 | 10.64 (6.94, 14.34) | < 0.001* | 1.70 (-0.95, 4.35) | 0.208 |
|  | Year 3 | 33.7 ± 9.6 | 2.58 (-1.05, 6.20) | 0.244 | 32.2 ± 10.1 | 7.59 (3.96, 11.21) | < 0.001* | 1.49 (-1.22, 4.20) | 0.282 |
| **Calcium** | Baseline | 9.1 ± 0.8 | Reference |  | 9.4 ± 0.7 | Reference |  | -0.37 (-0.58, -0.17) | < 0.001* |
|  | Six months | 9.5 ± 0.6 | 0.45 (0.21, 0.69) | < 0.001* | 9.2 ± 0.7 | -0.19 (-0.42, 0.04) | 0.130 | 0.27 (0.10, 0.44) | 0.002* |
|  | Year 1 | 9.3 ± 0.6 | 0.25 (0.01, 0.48) | 0.036* | 9.3 ± 0.8 | -0.15 (-0.40, 0.09) | 0.349 | 0.03 (-0.15, 0.20) | 0.771 |
|  | Year 2 | 9.5 ± 0.6 | 0.42 (0.18, 0.65) | < 0.001* | 9.5 ± 0.5 | 0.07 (-0.14, 0.28) | 0.788 | -0.02 (-0.17, 0.12) | 0.745 |
|  | Year 3 | 8.9 ± 0.5 | -0.12 (-0.36, 0.11) | 0.493 | 8.8 ± 0.5 | -0.57 (-0.78, -0.37) | < 0.001* | 0.08 (-0.07, 0.22) | 0.287 |
| **Triglycerides** | Baseline | 152.6 ± 50.5 | Reference |  | 141.7 ± 56.7 | Reference |  | 10.90 (-3.26, 25.07) | 0.131 |
|  | Six months | 123.7 ± 37.1 | -28.95 (-43.70, -14.21) | < 0.001* | 135.6 ± 36.4 | -6.17 (-21.81, 9.48) | 0.703 | -11.88 (-21.59, -2.17) | 0.017* |
|  | Year 1 | 115.6 ± 35.0 | -37.07 (-51.58, -22.55) | < 0.001* | 103.0 ± 35.4 | -38.73 (-54.30, -23.15) | < 0.001* | 12.56 (3.15, 21.98) | 0.009* |
|  | Year 2 | 90.7 ± 18.6 | -62.00 (-74.73, -49.26) | < 0.001* | 96.5 ± 25.3 | -45.19 (-59.71, -30.68) | < 0.001* | -5.90 (-12.01, 0.21) | 0.059 |
|  | Year 3 | 103.1 ± 27.4 | -49.55 (-63.22, -35.87) | < 0.001* | 97.9 ± 24.5 | -43.81 (-58.25, -29.38) | < 0.001* | 5.17 (-2.01, 12.35) | 0.158 |
| **Cholesterol** | Baseline | 180.5 ± 39.1 | Reference |  | 181.1 ± 41.7 | Reference |  | -0.60 (-11.27, 10.07) | 0.912 |
|  | Six months | 127.7 ± 44.5 | -52.86 (-66.79, -38.93) | < 0.001* | 119.0 ± 46.3 | -62.17 (-76.63, -47.70) | < 0.001* | 8.70 (-3.27, 20.68) | 0.154 |
|  | Year 1 | 129.6 ± 37.2 | -50.87 (-63.65, -38.10) | < 0.001* | 141.4 ± 33.3 | -39.71 (-52.17, -27.25) | < 0.001* | -11.76 (-21.21, -2.31) | 0.015* |
|  | Year 2 | 108.4 ± 33.1 | -72.11 (-84.42, -59.80) | < 0.001* | 98.3 ± 36.2 | -82.86 (-95.93, -69.78) | < 0.001* | 10.14 (0.56, 19.72) | 0.038* |
|  | Year 3 | 108.7 ± 40.2 | -71.80 (-85.34, -58.27) | < 0.001* | 99.5 ± 36.8 | -81.61 (-94.78, -68.45) | < 0.001* | 9.20 (-1.45, 19.86) | 0.090 |
| **LDL** | Baseline | 106.2 ± 26.8 | Reference |  | 96.3 ± 29.0 | Reference |  | 9.95 (2.58, 17.31) | 0.008* |
|  | Six months | 94.1 ± 19.9 | -12.09 (-19.94, -4.23) | 0.001* | 100.3 ± 23.7 | 4.01 (-4.69, 12.72) | 0.599 | -6.15 (-11.93, -0.37) | 0.037* |
|  | Year 1 | 98.2 ± 21.5 | -8.00 (-16.12, 0.11) | 0.055 | 100.8 ± 23.5 | 4.52 (-4.20, 13.23) | 0.508 | -2.57 (-8.59, 3.45) | 0.402 |
|  | Year 2 | 64.5 ± 16.2 | -41.71 (-49.17, -34.25) | < 0.001* | 70.6 ± 19.4 | -25.67 (-33.89, -17.46) | < 0.001* | -6.09 (-11.01, -1.16) | 0.015* |
|  | Year 3 | 76.8 ± 21.0 | -29.41 (-37.56, -21.25) | < 0.001* | 78.0 ± 20.9 | -18.32 (-26.75, -9.89) | < 0.001* | -1.14 (-6.93, 4.65) | 0.699 |
| **Fasting glucose** | Baseline | 96.1 ± 23.9 | Reference |  | 96.1 ± 20.1 | Reference |  | 0.03 (-5.80, 5.85) | 0.993 |
|  | Six months | 93.5 ± 9.4 | -2.55 (-8.58, 3.49) | 0.660 | 88.2 ± 12.3 | -7.87 (-13.33, -2.41) | 0.002* | 5.35 (2.47, 8.22) | < 0.001* |
|  | Year 1 | 85.7 ± 9.2 | -10.37 (-16.40, -4.34) | < 0.001* | 83.1 ± 11.6 | -12.95 (-18.35, -7.55) | < 0.001* | 2.61 (-0.19, 5.41) | 0.068 |
|  | Year 2 | 75.2 ± 7.3 | -20.85 (-26.74, -14.95) | < 0.001* | 80.0 ± 11.2 | -16.11 (-21.49, -10.72) | < 0.001* | -4.72 (-7.31, -2.12) | < 0.001* |
|  | Year 3 | 80.5 ± 11.1 | -15.59 (-21.84, -9.34) | < 0.001* | 79.2 ± 11.6 | -16.87 (-22.30, -11.43) | < 0.001* | 1.30 (-1.82, 4.43) | 0.414 |
| **HBA1C** | Baseline | 5.3 ± 0.6 | Reference |  | 5.3 ± 0.5 | Reference |  | 0.03 (-0.12, 0.18) | 0.664 |
|  | Six months | 4.9 ± 0.6 | -0.44 (-0.64, -0.24) | < 0.001* | 4.7 ± 0.6 | -0.63 (-0.82, -0.43) | < 0.001* | 0.22 (0.05, 0.38) | 0.011* |
|  | Year 1 | 5.0 ± 0.5 | -0.36 (-0.54, -0.18) | < 0.001* | 5.0 ± 0.5 | -0.28 (-0.45, -0.11) | < 0.001* | -0.05 (-0.18, 0.07) | 0.423 |
|  | Year 2 | 4.6 ± 0.5 | -0.76 (-0.95, -0.58) | < 0.001* | 4.6 ± 0.5 | -0.73 (-0.90, -0.55) | < 0.001* | 0.00 (-0.14, 0.13) | 0.965 |
|  | Year 3 | 4.5 ± 0.5 | -0.81 (-0.99, -0.63) | < 0.001* | 4.5 ± 0.5 | -0.85 (-1.03, -0.68) | < 0.001* | 0.07 (-0.06, 0.21) | 0.275 |
| **AST** | Baseline | 21.2 ± 9.3 | Reference |  | 21.3 ± 13.2 | Reference |  | -0.13 (-3.14, 2.87) | 0.930 |
|  | Six months | 27.0 ± 5.5 | 5.85 (3.30, 8.41) | < 0.001* | 25.1 ± 7.4 | 3.78 (0.27, 7.29) | 0.030* | 1.94 (0.22, 3.66) | 0.027* |
|  | Year 1 | 22.9 ± 7.0 | 1.71 (-1.05, 4.47) | 0.358 | 22.9 ± 7.8 | 1.60 (-1.97, 5.17) | 0.620 | -0.03 (-2.01, 1.96) | 0.980 |
|  | Year 2 | 25.5 ± 5.5 | 4.38 (1.80, 6.95) | < 0.001* | 25.5 ± 5.9 | 4.22 (0.85, 7.59) | 0.008* | 0.02 (-1.55, 1.59) | 0.978 |
|  | Year 3 | 25.4 ± 6.3 | 4.26 (1.58, 6.94) | < 0.001* | 26.4 ± 6.4 | 5.07 (1.64, 8.51) | 0.001* | -0.94 (-2.70, 0.81) | 0.290 |
| **ALT** | Baseline | 23.0 ± 13.3 | Reference |  | 22.9 ± 14.4 | Reference |  | 0.13 (-3.52, 3.79) | 0.943 |
|  | Six months | 24.7 ± 7.6 | 1.71 (-1.90, 5.32) | 0.578 | 25.8 ± 8.7 | 2.98 (-0.93, 6.89) | 0.192 | -1.14 (-3.30, 1.02) | 0.302 |
|  | Year 1 | 22.6 ± 9.9 | -0.42 (-4.33, 3.50) | 0.985 | 21.6 ± 11.0 | -1.26 (-5.49, 2.98) | 0.837 | 0.97 (-1.82, 3.77) | 0.495 |
|  | Year 2 | 30.8 ± 11.3 | 7.80 (3.60, 11.99) | < 0.001* | 29.0 ± 9.8 | 6.14 (2.04, 10.24) | 0.001* | 1.79 (-1.14, 4.71) | 0.231 |
|  | Year 3 | 25.1 ± 8.0 | 2.13 (-1.57, 5.83) | 0.420 | 26.5 ± 9.1 | 3.68 (-0.32, 7.69) | 0.082 | -1.42 (-3.78, 0.95) | 0.239 |
| **UREA** | Baseline | 26.4 ± 7.4 | Reference |  | 27.0 ± 9.2 | Reference |  | -0.60 (-2.79, 1.59) | 0.589 |
|  | Six months | 30.2 ± 7.7 | 3.76 (1.25, 6.28) | 0.001* | 28.1 ± 8.9 | 1.14 (-1.82, 4.10) | 0.718 | 2.02 (-0.17, 4.22) | 0.071 |
|  | Year 1 | 25.8 ± 7.9 | -0.59 (-3.16, 1.97) | 0.907 | 25.1 ± 8.4 | -1.87 (-4.78, 1.03) | 0.324 | 0.68 (-1.51, 2.86) | 0.543 |
|  | Year 2 | 27.7 ± 6.3 | 1.33 (-0.99, 3.66) | 0.423 | 33.0 ± 8.0 | 5.95 (3.06, 8.83) | < 0.001* | -5.21 (-7.20, -3.23) | < 0.001* |
|  | Year 3 | 29.1 ± 7.1 | 2.68 (0.20, 5.15) | 0.029* | 28.5 ± 8.3 | 1.52 (-1.40, 4.44) | 0.503 | 0.55 (-1.58, 2.68) | 0.611 |
| **CRT** | Baseline | 0.7 ± 0.2 | Reference |  | 0.8 ± 0.2 | Reference |  | -0.06 (-0.11, -0.01) | 0.013* |
|  | Six months | 1.0 ± 0.2 | 0.20 (0.14, 0.26) | < 0.001* | 0.9 ± 0.2 | 0.12 (0.06, 0.19) | < 0.001* | 0.02 (-0.03, 0.07) | 0.535 |
|  | Year 1 | 1.0 ± 0.2 | 0.21 (0.15, 0.27) | < 0.001* | 0.9 ± 0.3 | 0.13 (0.05, 0.21) | < 0.001* | 0.02 (-0.04, 0.09) | 0.509 |
|  | Year 2 | 0.9 ± 0.2 | 0.15 (0.10, 0.20) | < 0.001* | 1.0 ± 0.2 | 0.22 (0.15, 0.28) | < 0.001* | -0.13 (-0.17, -0.09) | < 0.001* |
|  | Year 3 | 0.9 ± 0.3 | 0.14 (0.06, 0.21) | < 0.001* | 0.9 ± 0.2 | 0.10 (0.03, 0.18) | 0.003* | -0.03 (-0.10, 0.04) | 0.399 |
| **TSH** | Baseline | 1.9 ± 0.9 | Reference |  | 2.1 ± 1.0 | Reference |  | -0.18 (-0.44, 0.07) | 0.159 |
|  | Six months | 2.1 ± 0.9 | 0.16 (-0.14, 0.45) | 0.488 | 2.2 ± 1.4 | 0.12 (-0.28, 0.52) | 0.841 | -0.14 (-0.45, 0.16) | 0.354 |
|  | Year 1 | 2.1 ± 1.2 | 0.21 (-0.15, 0.56) | 0.418 | 1.7 ± 1.0 | -0.42 (-0.75, -0.08) | 0.008* | 0.44 (0.14, 0.74) | 0.004* |
|  | Year 2 | 2.3 ± 1.0 | 0.39 (0.07, 0.71) | 0.012* | 1.9 ± 1.0 | -0.19 (-0.54, 0.16) | 0.472 | 0.39 (0.11, 0.67) | 0.006* |
|  | Year 3 | 2.4 ± 1.0 | 0.51 (0.18, 0.84) | 0.001* | 2.4 ± 1.0 | 0.34 (-0.01, 0.68) | 0.056 | -0.01 (-0.30, 0.27) | 0.920 |
| **FT3** | Baseline | 3.2 ± 0.5 | Reference |  | 3.2 ± 0.6 | Reference |  | -0.04 (-0.18, 0.10) | 0.596 |
|  | Six months | 2.8 ± 0.7 | -0.41 (-0.62, -0.20) | < 0.001* | 3.0 ± 0.6 | -0.27 (-0.45, -0.08) | 0.002* | -0.18 (-0.36, -0.01) | 0.040* |
|  | Year 1 | 3.1 ± 0.7 | -0.08 (-0.30, 0.13) | 0.703 | 3.1 ± 0.7 | -0.13 (-0.35, 0.08) | 0.355 | 0.01 (-0.18, 0.21) | 0.895 |
|  | Year 2 | 3.5 ± 0.7 | 0.28 (0.07, 0.49) | 0.005* | 3.2 ± 0.8 | -0.01 (-0.24, 0.21) | 0.996 | 0.25 (0.05, 0.46) | 0.016* |
|  | Year 3 | 3.1 ± 0.7 | -0.13 (-0.33, 0.08) | 0.356 | 3.3 ± 0.7 | 0.03 (-0.19, 0.25) | 0.966 | -0.20 (-0.40, 0.00) | 0.047* |
| **FT4** | Baseline | 1.1 ± 0.2 | Reference |  | 1.2 ± 0.2 | Reference |  | -0.07 (-0.12, -0.01) | 0.013* |
|  | Six months | 1.2 ± 0.2 | 0.09 (0.02, 0.17) | 0.008* | 1.4 ± 0.2 | 0.17 (0.10, 0.24) | < 0.001* | -0.14 (-0.20, -0.08) | < 0.001* |
|  | Year 1 | 1.3 ± 0.2 | 0.10 (0.03, 0.18) | 0.002* | 1.3 ± 0.2 | 0.06 (-0.01, 0.14) | 0.082 | -0.03 (-0.09, 0.03) | 0.377 |
|  | Year 2 | 1.2 ± 0.3 | 0.09 (0.02, 0.17) | 0.012* | 1.3 ± 0.2 | 0.07 (0.00, 0.14) | 0.036* | -0.05 (-0.11, 0.02) | 0.168 |
|  | Year 3 | 1.3 ± 0.2 | 0.11 (0.03, 0.18) | 0.002* | 1.3 ± 0.2 | 0.08 (0.01, 0.16) | 0.020* | -0.05 (-0.11, 0.02) | 0.169 |
| **HB** | Baseline | 12.9 ± 1.7 | Reference |  | 12.6 ± 1.3 | Reference |  | 0.31 (-0.09, 0.71) | 0.127 |
|  | Six months | 14.0 ± 1.9 | 1.08 (0.48, 1.67) | < 0.001* | 13.6 ± 1.4 | 1.05 (0.61, 1.48) | < 0.001* | 0.34 (-0.10, 0.78) | 0.125 |
|  | Year 1 | 12.7 ± 1.5 | -0.18 (-0.71, 0.34) | 0.768 | 13.3 ± 2.0 | 0.76 (0.20, 1.31) | 0.003* | -0.63 (-1.09, -0.17) | 0.008* |
|  | Year 2 | 13.1 ± 1.3 | 0.19 (-0.32, 0.70) | 0.732 | 13.0 ± 1.4 | 0.45 (-0.01, 0.91) | 0.060 | 0.05 (-0.33, 0.43) | 0.784 |
|  | Year 3 | 12.7 ± 1.8 | -0.16 (-0.76, 0.44) | 0.873 | 12.9 ± 1.6 | 0.35 (-0.13, 0.84) | 0.220 | -0.20 (-0.68, 0.27) | 0.394 |
| **Ferritin** | Baseline | 59.9 ± 65.5 | Reference |  | 30.4 ± 12.8 | Reference |  | 29.51 (16.98, 42.04) | < 0.001* |
|  | Six months | 60.3 ± 76.0 | 0.36 (-23.25, 23.98) | 1.000 | 30.1 ± 12.4 | -0.32 (-4.46, 3.81) | 0.993 | 30.20 (15.72, 44.67) | < 0.001* |
|  | Year 1 | 74.3 ± 86.2 | 14.42 (-11.28, 40.11) | 0.442 | 45.4 ± 53.8 | 15.03 (2.02, 28.03) | 0.017* | 28.90 (9.62, 48.18) | 0.003* |
|  | Year 2 | 125.1 ± 45.6 | 65.22 (46.13, 84.30) | < 0.001* | 90.5 ± 55.0 | 60.10 (46.45, 73.75) | < 0.001* | 34.63 (20.70, 48.56) | < 0.001* |
|  | Year 3 | 74.4 ± 41.8 | 14.51 (-4.04, 33.05) | 0.174 | 71.8 ± 45.2 | 41.34 (30.01, 52.67) | < 0.001* | 2.67 (-9.33, 14.68) | 0.662 |
| **INR** | Baseline | 1.0 ± 0.0 | Reference |  | 1.0 ± 0.1 | Reference |  | -0.01 (-0.02, 0.01) | 0.333 |
|  | Six months | 1.0 ± 0.0 | -0.02 (-0.03, -0.01) | < 0.001* | 1.0 ± 0.0 | -0.03 (-0.04, -0.01) | < 0.001* | 0.00 (0.00, 0.00) | 0.378 |
|  | Year 1 | 1.0 ± 0.0 | -0.02 (-0.03, -0.01) | < 0.001* | 1.0 ± 0.0 | -0.03 (-0.04, -0.01) | < 0.001* | 0.00 (-0.01, 0.00) | 0.211 |
|  | Year 2 | 1.0 ± 0.0 | -0.02 (-0.03, 0.00) | 0.008* | 1.0 ± 0.0 | -0.03 (-0.04, -0.01) | 0.001* | 0.00 (0.00, 0.01) | 0.512 |
|  | Year 3 | 1.0 ± 0.0 | -0.02 (-0.03, -0.01) | < 0.001* | 1.0 ± 0.0 | -0.02 (-0.04, -0.01) | 0.001* | 0.00 (0.00, 0.00) | 0.582 |
| **WHITE CELL COUNT** | Baseline | 7.1 ± 2.3 | Reference |  | 7.7 ± 2.4 | Reference |  | -0.64 (-1.26, -0.02) | 0.043* |
|  | Six months | 7.5 ± 1.9 | 0.47 (-0.23, 1.17) | 0.286 | 7.0 ± 2.3 | -0.72 (-1.49, 0.06) | 0.080 | 0.55 (-0.01, 1.10) | 0.054 |
|  | Year 1 | 6.2 ± 1.7 | -0.84 (-1.50, -0.17) | 0.008* | 6.7 ± 1.7 | -1.03 (-1.73, -0.33) | 0.001* | -0.45 (-0.90, 0.00) | 0.051 |
|  | Year 2 | 4.9 ± 0.8 | -2.14 (-2.70, -1.57) | < 0.001* | 5.7 ± 1.7 | -2.07 (-2.77, -1.37) | < 0.001* | -0.71 (-1.06, -0.36) | < 0.001* |
|  | Year 3 | 6.1 ± 1.9 | -1.02 (-1.72, -0.32) | 0.002* | 6.1 ± 2.0 | -1.57 (-2.32, -0.83) | < 0.001* | -0.09 (-0.62, 0.44) | 0.744 |

Cell values represent Mean ± standard deviations. *Statistically significant (p < 0.05). MD: Mean difference. CI: Confidence Interval. HB = Hemoglobin; AST = aspartate aminotransferase; ALT = alanine aminotransferase; INR = international normalized ratio; FT3 = free triiodothyronine; FT4 = free thyroxine; TSH = thyroid-stimulating hormone; LDL = low‑density lipoprotein; HbA1c = glycated hemoglobin.

| **Table S9: Lab results’ estimates from GEE analyses for the within and between the SG and Ra-SG groups (Complete cases analysis)** | | | | | | | | | |
| --- | --- | --- | --- | --- | --- | --- | --- | --- | --- |
| **Variable** | **Time** | **SG** | | | **Ra-SG** | | | **MD between SG and Ra-SG(95% CI)** | **p** |
|  |  | **M ± SD** | **MD from baseline** | **p** | **M ± SD** | **MD from baseline** | **p** |  |  |
| **Albumin** | Baseline | 4.5 ± 0.6 | Reference |  | 4.4 ± 0.5 | Reference |  | 0.06 (-0.09, 0.22) | 0.422 |
|  | Six months | 4.4 ± 0.5 | -0.09 (-0.29, 0.11) | 0.594 | 4.5 ± 0.6 | 0.10 (-0.10, 0.30) | 0.531 | -0.13 (-0.29, 0.03) | 0.117 |
|  | Year 1 | 4.2 ± 0.5 | -0.30 (-0.48, -0.11) | < 0.001* | 4.3 ± 0.5 | -0.13 (-0.30, 0.05) | 0.222 | -0.10 (-0.24, 0.03) | 0.116 |
|  | Year 2 | 4.3 ± 0.4 | -0.21 (-0.38, -0.03) | 0.013* | 4.4 ± 0.5 | 0.03 (-0.14, 0.20) | 0.945 | -0.18 (-0.29, -0.06) | 0.003* |
|  | Year 3 | 4.0 ± 0.4 | -0.44 (-0.61, -0.26) | < 0.001* | 4.0 ± 0.4 | -0.38 (-0.54, -0.22) | < 0.001* | 0.01 (-0.10, 0.12) | 0.883 |
| **VITAMIN B12** | Baseline | 608.2 ± 385.6 | Reference |  | 440.7 ± 129.2 | Reference |  | 167.44 (87.39, 247.49) | < 0.001* |
|  | Six months | 585.9 ± 412.4 | -22.25 (-161.68, 117.18) | 0.961 | 400.7 ± 147.2 | -40.04 (-87.46, 7.38) | 0.126 | 185.23 (99.06, 271.41) | < 0.001* |
|  | Year 1 | 532.8 ± 299.7 | -75.35 (-195.96, 45.25) | 0.351 | 529.1 ± 353.3 | 88.38 (-2.68, 179.45) | 0.060 | 3.71 (-86.62, 94.03) | 0.936 |
|  | Year 2 | 654.4 ± 228.7 | 46.24 (-64.47, 156.95) | 0.667 | 555.0 ± 219.3 | 114.27 (52.64, 175.90) | < 0.001* | 99.41 (37.50, 161.31) | 0.002* |
|  | Year 3 | 591.5 ± 159.1 | -16.71 (-119.71, 86.30) | 0.960 | 543.8 ± 163.1 | 103.01 (52.63, 153.39) | < 0.001* | 47.72 (3.24, 92.20) | 0.035* |
| **Vit D** | Baseline | 31.3 ± 12.2 | Reference |  | 25.5 ± 11.4 | Reference |  | 5.81 (2.54, 9.08) | 0.001* |
|  | Six months | 25.2 ± 11.4 | -6.05 (-10.18, -1.92) | 0.001* | 34.6 ± 11.7 | 9.07 (5.10, 13.03) | < 0.001* | -9.31 (-12.50, -6.11) | < 0.001* |
|  | Year 1 | 26.7 ± 9.6 | -4.55 (-8.39, -0.71) | 0.014* | 31.1 ± 15.2 | 5.65 (1.04, 10.25) | 0.010* | -4.39 (-7.88, -0.89) | 0.014* |
|  | Year 2 | 36.9 ± 8.6 | 5.63 (1.94, 9.32) | 0.001* | 35.2 ± 10.5 | 9.74 (5.97, 13.50) | < 0.001* | 1.70 (-0.95, 4.35) | 0.208 |
|  | Year 3 | 33.7 ± 9.6 | 2.36 (-1.47, 6.19) | 0.362 | 32.2 ± 10.1 | 6.68 (2.99, 10.37) | < 0.001* | 1.49 (-1.22, 4.20) | 0.282 |
| **Calcium** | Baseline | 9.1 ± 0.8 | Reference |  | 9.4 ± 0.7 | Reference |  | -0.32 (-0.53, -0.11) | 0.003* |
|  | Six months | 9.5 ± 0.6 | 0.37 (0.10, 0.63) | 0.002* | 9.2 ± 0.7 | -0.20 (-0.43, 0.03) | 0.107 | 0.25 (0.07, 0.43) | 0.006* |
|  | Year 1 | 9.3 ± 0.5 | 0.15 (-0.10, 0.40) | 0.375 | 9.2 ± 0.7 | -0.20 (-0.44, 0.05) | 0.162 | 0.03 (-0.15, 0.21) | 0.759 |
|  | Year 2 | 9.5 ± 0.6 | 0.37 (0.12, 0.62) | 0.001* | 9.5 ± 0.5 | 0.07 (-0.13, 0.28) | 0.757 | -0.02 (-0.17, 0.12) | 0.745 |
|  | Year 3 | 8.9 ± 0.5 | -0.17 (-0.42, 0.07) | 0.250 | 8.8 ± 0.5 | -0.57 (-0.78, -0.36) | < 0.001* | 0.08 (-0.07, 0.22) | 0.287 |
| **Triglycerides** | Baseline | 152.6 ± 51.3 | Reference |  | 140.3 ± 56.8 | Reference |  | 12.29 (-2.64, 27.23) | 0.107 |
|  | Six months | 126.8 ± 36.2 | -25.79 (-41.29, -10.29) | < 0.001* | 134.0 ± 37.3 | -6.24 (-22.69, 10.21) | 0.725 | -7.25 (-17.39, 2.89) | 0.161 |
|  | Year 1 | 114.6 ± 34.3 | -37.92 (-53.16, -22.68) | < 0.001* | 103.0 ± 35.9 | -37.23 (-53.50, -20.97) | < 0.001* | 11.60 (1.92, 21.29) | 0.019* |
|  | Year 2 | 90.7 ± 18.6 | -61.90 (-75.37, -48.42) | < 0.001* | 96.5 ± 25.3 | -43.71 (-58.76, -28.66) | < 0.001* | -5.90 (-12.01, 0.21) | 0.059 |
|  | Year 3 | 103.1 ± 27.4 | -49.45 (-63.81, -35.08) | < 0.001* | 97.9 ± 24.5 | -42.33 (-57.30, -27.35) | < 0.001* | 5.17 (-2.01, 12.35) | 0.158 |
| **Cholesterol** | Baseline | 180.4 ± 40.7 | Reference |  | 181.1 ± 42.7 | Reference |  | -0.64 (-12.16, 10.87) | 0.913 |
|  | Six months | 129.4 ± 43.1 | -51.05 (-65.69, -36.41) | < 0.001* | 117.7 ± 47.2 | -63.42 (-78.83, -48.00) | < 0.001* | 11.72 (-0.75, 24.20) | 0.066 |
|  | Year 1 | 128.3 ± 37.4 | -52.12 (-65.77, -38.47) | < 0.001* | 141.4 ± 33.5 | -39.72 (-52.85, -26.58) | < 0.001* | -13.05 (-22.86, -3.24) | 0.009* |
|  | Year 2 | 108.4 ± 33.1 | -72.04 (-85.00, -59.09) | < 0.001* | 98.3 ± 36.2 | -82.83 (-96.39, -69.26) | < 0.001* | 10.14 (0.56, 19.72) | 0.038* |
|  | Year 3 | 108.7 ± 40.2 | -71.73 (-85.87, -57.60) | < 0.001* | 99.5 ± 36.8 | -81.58 (-95.23, -67.93) | < 0.001* | 9.20 (-1.46, 19.86) | 0.090 |
| **LDL** | Baseline | 103.8 ± 26.7 | Reference |  | 95.3 ± 29.8 | Reference |  | 8.54 (0.73, 16.34) | 0.032* |
|  | Six months | 96.3 ± 18.9 | -7.51 (-15.58, 0.57) | 0.078 | 98.8 ± 24.1 | 3.46 (-5.83, 12.74) | 0.736 | -2.42 (-8.40, 3.55) | 0.426 |
|  | Year 1 | 97.8 ± 21.2 | -6.00 (-14.42, 2.41) | 0.241 | 100.2 ± 23.7 | 4.93 (-4.29, 14.15) | 0.482 | -2.40 (-8.60, 3.80) | 0.448 |
|  | Year 2 | 64.5 ± 16.2 | -39.32 (-47.03, -31.61) | < 0.001* | 70.6 ± 19.4 | -24.70 (-33.30, -16.09) | < 0.001* | -6.09 (-11.01, -1.16) | 0.015* |
|  | Year 3 | 76.8 ± 21.0 | -27.02 (-35.41, -18.64) | < 0.001* | 78.0 ± 20.9 | -17.34 (-26.16, -8.53) | < 0.001* | -1.14 (-6.93, 4.65) | 0.699 |
| **Fasting glucose** | Baseline | 96.5 ± 24.9 | Reference |  | 96.2 ± 20.3 | Reference |  | 0.26 (-6.03, 6.55) | 0.935 |
|  | Six months | 94.4 ± 9.4 | -2.11 (-8.69, 4.48) | 0.808 | 88.8 ± 12.1 | -7.39 (-13.10, -1.67) | 0.006* | 5.54 (2.56, 8.53) | < 0.001* |
|  | Year 1 | 85.7 ± 9.1 | -10.75 (-17.30, -4.19) | < 0.001* | 82.7 ± 11.4 | -13.49 (-19.11, -7.87) | < 0.001* | 3.00 (0.17, 5.84) | 0.038* |
|  | Year 2 | 75.2 ± 7.3 | -21.23 (-27.64, -14.81) | < 0.001* | 80.0 ± 11.2 | -16.25 (-21.85, -10.65) | < 0.001* | -4.72 (-7.31, -2.12) | < 0.001* |
|  | Year 3 | 80.5 ± 11.1 | -15.97 (-22.70, -9.23) | < 0.001* | 79.2 ± 11.6 | -17.01 (-22.66, -11.36) | < 0.001* | 1.30 (-1.83, 4.43) | 0.414 |
| **HBA1C** | Baseline | 5.3 ± 0.6 | Reference |  | 5.3 ± 0.6 | Reference |  | 0.02 (-0.14, 0.19) | 0.768 |
|  | Six months | 5.0 ± 0.6 | -0.36 (-0.57, -0.14) | < 0.001* | 4.7 ± 0.7 | -0.62 (-0.82, -0.41) | < 0.001* | 0.29 (0.11, 0.46) | 0.001* |
|  | Year 1 | 5.0 ± 0.5 | -0.36 (-0.55, -0.17) | < 0.001* | 5.0 ± 0.5 | -0.29 (-0.46, -0.11) | < 0.001* | -0.05 (-0.18, 0.08) | 0.442 |
|  | Year 2 | 4.6 ± 0.5 | -0.75 (-0.95, -0.56) | < 0.001* | 4.6 ± 0.5 | -0.73 (-0.90, -0.55) | < 0.001* | 0.00 (-0.14, 0.13) | 0.965 |
|  | Year 3 | 4.5 ± 0.5 | -0.80 (-0.99, -0.61) | < 0.001* | 4.5 ± 0.5 | -0.85 (-1.04, -0.67) | < 0.001* | 0.07 (-0.06, 0.21) | 0.275 |
| **AST** | Baseline | 21.2 ± 9.2 | Reference |  | 21.2 ± 13.3 | Reference |  | -0.03 (-3.18, 3.12) | 0.987 |
|  | Six months | 27.3 ± 5.5 | 6.08 (3.43, 8.72) | < 0.001* | 24.6 ± 7.3 | 3.40 (-0.28, 7.07) | 0.080 | 2.65 (0.87, 4.44) | 0.004* |
|  | Year 1 | 23.0 ± 6.9 | 1.74 (-1.09, 4.58) | 0.364 | 22.3 ± 7.6 | 1.06 (-2.66, 4.77) | 0.852 | 0.66 (-1.34, 2.67) | 0.518 |
|  | Year 2 | 25.5 ± 5.5 | 4.32 (1.68, 6.96) | < 0.001* | 25.5 ± 5.9 | 4.27 (0.74, 7.80) | 0.011* | 0.02 (-1.55, 1.59) | 0.978 |
|  | Year 3 | 25.4 ± 6.3 | 4.21 (1.46, 6.95) | 0.001* | 26.4 ± 6.4 | 5.12 (1.54, 8.71) | 0.002* | -0.94 (-2.70, 0.81) | 0.290 |
| **ALT** | Baseline | 23.0 ± 13.5 | Reference |  | 22.7 ± 14.5 | Reference |  | 0.34 (-3.52, 4.20) | 0.861 |
|  | Six months | 24.6 ± 7.9 | 1.54 (-2.32, 5.40) | 0.697 | 25.0 ± 8.6 | 2.30 (-1.77, 6.38) | 0.436 | -0.42 (-2.70, 1.86) | 0.718 |
|  | Year 1 | 22.4 ± 9.8 | -0.59 (-4.71, 3.53) | 0.970 | 21.7 ± 11.2 | -1.03 (-5.46, 3.40) | 0.907 | 0.78 (-2.13, 3.69) | 0.598 |
|  | Year 2 | 30.8 ± 11.3 | 7.76 (3.41, 12.10) | < 0.001* | 29.0 ± 9.8 | 6.31 (2.08, 10.54) | 0.001* | 1.79 (-1.14, 4.71) | 0.231 |
|  | Year 3 | 25.1 ± 8.0 | 2.09 (-1.78, 5.96) | 0.474 | 26.5 ± 9.1 | 3.85 (-0.29, 7.99) | 0.077 | -1.42 (-3.78, 0.95) | 0.239 |
| **UREA** | Baseline | 26.5 ± 7.5 | Reference |  | 27.4 ± 9.3 | Reference |  | -0.92 (-3.26, 1.42) | 0.442 |
|  | Six months | 30.0 ± 8.1 | 3.50 (0.77, 6.23) | 0.006* | 28.7 ± 9.0 | 1.29 (-1.85, 4.43) | 0.677 | 1.29 (-1.07, 3.65) | 0.283 |
|  | Year 1 | 26.3 ± 8.0 | -0.24 (-2.95, 2.47) | 0.991 | 25.5 ± 8.5 | -1.97 (-5.03, 1.10) | 0.327 | 0.81 (-1.47, 3.10) | 0.484 |
|  | Year 2 | 27.7 ± 6.3 | 1.22 (-1.20, 3.64) | 0.530 | 33.0 ± 8.0 | 5.52 (2.53, 8.50) | < 0.001* | -5.21 (-7.20, -3.23) | < 0.001* |
|  | Year 3 | 29.1 ± 7.1 | 2.56 (0.00, 5.13) | 0.050* | 28.5 ± 8.3 | 1.10 (-1.93, 4.12) | 0.750 | 0.55 (-1.58, 2.68) | 0.611 |
| **Creatinine** | Baseline | 0.7 ± 0.2 | Reference |  | 0.8 ± 0.2 | Reference |  | -0.07 (-0.12, -0.01) | 0.012* |
|  | Six months | 0.9 ± 0.2 | 0.20 (0.14, 0.26) | < 0.001* | 0.9 ± 0.2 | 0.12 (0.05, 0.19) | < 0.001* | 0.01 (-0.04, 0.07) | 0.623 |
|  | Year 1 | 1.0 ± 0.2 | 0.22 (0.15, 0.28) | < 0.001* | 0.9 ± 0.3 | 0.13 (0.05, 0.21) | 0.001* | 0.02 (-0.04, 0.09) | 0.515 |
|  | Year 2 | 0.9 ± 0.2 | 0.15 (0.10, 0.21) | < 0.001* | 1.0 ± 0.2 | 0.21 (0.15, 0.28) | < 0.001* | -0.13 (-0.17, -0.09) | < 0.001* |
|  | Year 3 | 0.9 ± 0.3 | 0.14 (0.06, 0.21) | < 0.001* | 0.9 ± 0.2 | 0.10 (0.02, 0.18) | 0.006* | -0.03 (-0.10, 0.04) | 0.399 |
| **TSH** | Baseline | 1.9 ± 0.9 | Reference |  | 2.2 ± 1.0 | Reference |  | -0.22 (-0.49, 0.05) | 0.112 |
|  | Six months | 2.2 ± 0.9 | 0.23 (-0.08, 0.55) | 0.205 | 2.2 ± 1.4 | 0.01 (-0.41, 0.44) | 0.999 | 0.00 (-0.32, 0.33) | 0.986 |
|  | Year 1 | 2.2 ± 1.2 | 0.22 (-0.16, 0.60) | 0.418 | 1.7 ± 1.0 | -0.46 (-0.81, -0.10) | 0.006* | 0.46 (0.14, 0.77) | 0.004* |
|  | Year 2 | 2.3 ± 1.0 | 0.38 (0.04, 0.71) | 0.020* | 1.9 ± 1.0 | -0.24 (-0.59, 0.12) | 0.298 | 0.39 (0.11, 0.67) | 0.006* |
|  | Year 3 | 2.4 ± 1.0 | 0.50 (0.15, 0.84) | 0.002* | 2.4 ± 1.0 | 0.29 (-0.06, 0.65) | 0.145 | -0.01 (-0.30, 0.27) | 0.920 |
| **FT3** | Baseline | 3.2 ± 0.5 | Reference |  | 3.2 ± 0.5 | Reference |  | -0.05 (-0.20, 0.09) | 0.486 |
|  | Six months | 2.8 ± 0.7 | -0.32 (-0.54, -0.10) | 0.002* | 3.0 ± 0.6 | -0.21 (-0.40, -0.01) | 0.032* | -0.16 (-0.35, 0.02) | 0.087 |
|  | Year 1 | 3.1 ± 0.7 | -0.05 (-0.27, 0.17) | 0.904 | 3.1 ± 0.7 | -0.14 (-0.36, 0.09) | 0.371 | 0.03 (-0.17, 0.24) | 0.752 |
|  | Year 2 | 3.5 ± 0.7 | 0.31 (0.09, 0.52) | 0.002* | 3.2 ± 0.8 | 0.00 (-0.23, 0.23) | 1.000 | 0.25 (0.05, 0.46) | 0.016* |
|  | Year 3 | 3.1 ± 0.7 | -0.10 (-0.31, 0.11) | 0.590 | 3.3 ± 0.7 | 0.05 (-0.17, 0.27) | 0.917 | -0.20 (-0.40, 0.00) | 0.047* |
| **FT4** | Baseline | 1.1 ± 0.2 | Reference |  | 1.2 ± 0.2 | Reference |  | -0.07 (-0.13, -0.01) | 0.015* |
|  | Six months | 1.2 ± 0.2 | 0.08 (0.01, 0.16) | 0.027* | 1.4 ± 0.2 | 0.18 (0.11, 0.25) | < 0.001* | -0.16 (-0.23, -0.10) | < 0.001* |
|  | Year 1 | 1.2 ± 0.2 | 0.10 (0.02, 0.18) | 0.009* | 1.3 ± 0.2 | 0.07 (-0.01, 0.14) | 0.090 | -0.04 (-0.10, 0.02) | 0.231 |
|  | Year 2 | 1.2 ± 0.3 | 0.09 (0.01, 0.18) | 0.015* | 1.3 ± 0.2 | 0.07 (0.00, 0.14) | 0.052 | -0.05 (-0.11, 0.02) | 0.168 |
|  | Year 3 | 1.3 ± 0.2 | 0.11 (0.03, 0.18) | 0.003* | 1.3 ± 0.2 | 0.08 (0.01, 0.16) | 0.030* | -0.05 (-0.11, 0.02) | 0.169 |
| **HB** | Baseline | 12.8 ± 1.7 | Reference |  | 12.5 ± 1.3 | Reference |  | 0.31 (-0.11, 0.73) | 0.152 |
|  | Six months | 13.7 ± 1.8 | 0.90 (0.28, 1.52) | 0.001* | 13.6 ± 1.3 | 1.07 (0.62, 1.53) | < 0.001* | 0.13 (-0.31, 0.58) | 0.559 |
|  | Year 1 | 12.7 ± 1.4 | -0.18 (-0.74, 0.37) | 0.794 | 13.3 ± 2.0 | 0.80 (0.23, 1.38) | 0.003* | -0.68 (-1.16, -0.20) | 0.006* |
|  | Year 2 | 13.1 ± 1.3 | 0.24 (-0.30, 0.77) | 0.628 | 13.0 ± 1.4 | 0.49 (0.02, 0.96) | 0.038* | 0.05 (-0.33, 0.43) | 0.784 |
|  | Year 3 | 12.7 ± 1.8 | -0.12 (-0.74, 0.51) | 0.944 | 12.9 ± 1.6 | 0.40 (-0.09, 0.89) | 0.152 | -0.20 (-0.68, 0.27) | 0.394 |
| **Ferritin** | Baseline | 63.1 ± 68.0 | Reference |  | 30.7 ± 13.2 | Reference |  | 32.37 (18.73, 46.02) | < 0.001* |
|  | Six months | 62.4 ± 77.1 | -0.70 (-26.07, 24.67) | 0.999 | 30.9 ± 12.6 | 0.13 (-4.29, 4.55) | 0.999 | 31.54 (16.15, 46.93) | < 0.001* |
|  | Year 1 | 76.7 ± 88.5 | 13.57 (-13.99, 41.14) | 0.549 | 46.6 ± 55.1 | 15.83 (2.13, 29.54) | 0.017* | 30.11 (9.66, 50.56) | 0.004* |
|  | Year 2 | 125.1 ± 45.6 | 62.04 (41.82, 82.25) | < 0.001* | 90.5 ± 55.0 | 59.78 (46.07, 73.48) | < 0.001* | 34.63 (20.70, 48.56) | < 0.001* |
|  | Year 3 | 74.4 ± 41.8 | 11.33 (-8.38, 31.03) | 0.422 | 71.8 ± 45.2 | 41.02 (29.63, 52.42) | < 0.001* | 2.67 (-9.33, 14.68) | 0.662 |
| **INR** | Baseline | 1.0 ± 0.0 | Reference |  | 1.0 ± 0.1 | Reference |  | -0.01 (-0.02, 0.01) | 0.536 |
|  | Six months | 1.0 ± 0.0 | -0.02 (-0.03, -0.01) | < 0.001* | 1.0 ± 0.0 | -0.03 (-0.04, -0.01) | 0.001* | 0.00 (0.00, 0.00) | 0.378 |
|  | Year 1 | 1.0 ± 0.0 | -0.02 (-0.03, -0.01) | < 0.001* | 1.0 ± 0.0 | -0.02 (-0.04, -0.01) | 0.002* | 0.00 (-0.01, 0.00) | 0.210 |
|  | Year 2 | 1.0 ± 0.0 | -0.02 (-0.03, 0.00) | 0.004* | 1.0 ± 0.0 | -0.02 (-0.04, -0.01) | 0.003* | 0.00 (0.00, 0.01) | 0.512 |
|  | Year 3 | 1.0 ± 0.0 | -0.02 (-0.03, -0.01) | < 0.001* | 1.0 ± 0.0 | -0.02 (-0.04, -0.01) | 0.004* | 0.00 (0.00, 0.00) | 0.582 |
| **WHITE CELL COUNT** | Baseline | 7.1 ± 2.3 | Reference |  | 7.8 ± 2.5 | Reference |  | -0.72 (-1.38, -0.06) | 0.031* |
|  | Six months | 7.6 ± 2.0 | 0.54 (-0.20, 1.29) | 0.227 | 6.9 ± 2.2 | -0.92 (-1.72, -0.12) | 0.019* | 0.75 (0.16, 1.33) | 0.012* |
|  | Year 1 | 6.3 ± 1.6 | -0.83 (-1.52, -0.14) | 0.011* | 6.7 ± 1.7 | -1.08 (-1.80, -0.36) | 0.001* | -0.47 (-0.93, -0.02) | 0.041* |
|  | Year 2 | 4.9 ± 0.8 | -2.15 (-2.74, -1.55) | < 0.001* | 5.7 ± 1.7 | -2.16 (-2.87, -1.44) | < 0.001* | -0.71 (-1.06, -0.36) | < 0.001* |
|  | Year 3 | 6.1 ± 1.9 | -1.03 (-1.76, -0.30) | 0.002* | 6.1 ± 2.0 | -1.66 (-2.43, -0.90) | < 0.001* | -0.09 (-0.62, 0.44) | 0.744 |

Cell values represent Mean ± standard deviations. *Statistically significant (p < 0.05). MD: Mean difference. CI: Confidence Interval

**Figure S1**


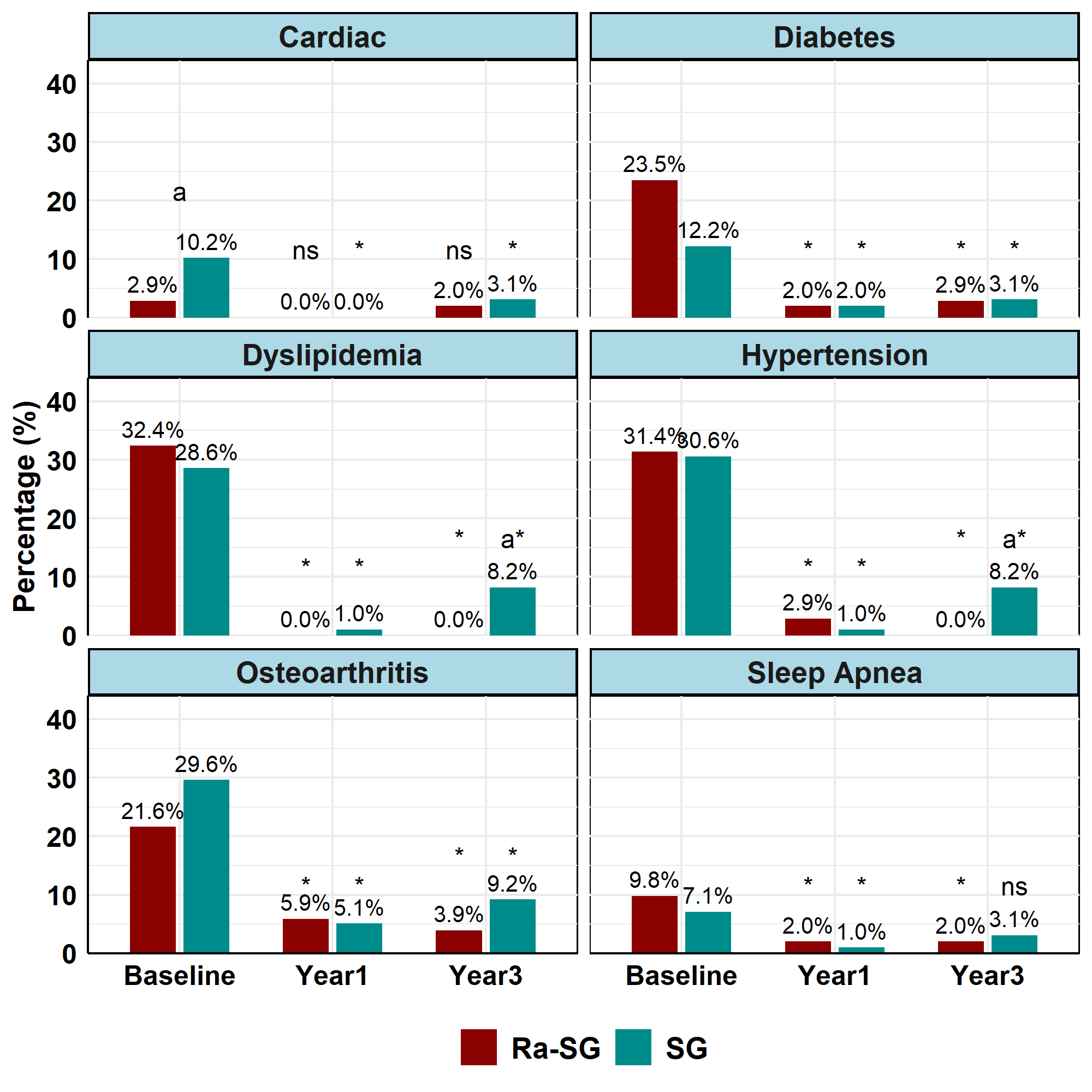


**Figure S1:** Trends in Resolution and Recurrence of obesity related diseases after SG vs. Ra-SG. This figure illustrates the changes in prevalence of key associated medical problems, including cardiac issues, diabetes, dyslipidemia, hypertension, osteoarthritis, and sleep apnea, across three time points: Baseline, Year 1, and Year 3 post-surgery. It presents a comparative view between patients undergoing SG and those undergoing Ra-SG. The percentages reflect the proportion of patients with each obesity related disease at the respective time points. Symbols on the bars represent significant changes: ‘a’ indicates a significant difference between SG and Ra-SG at a particular time point, '*' denotes significant changes from baseline within each group, and 'ns' indicates no significant change. The analysis underscores the effectiveness of both surgical interventions in managing and potentially resolving associated medical problems over the long term.

**Figure S2**


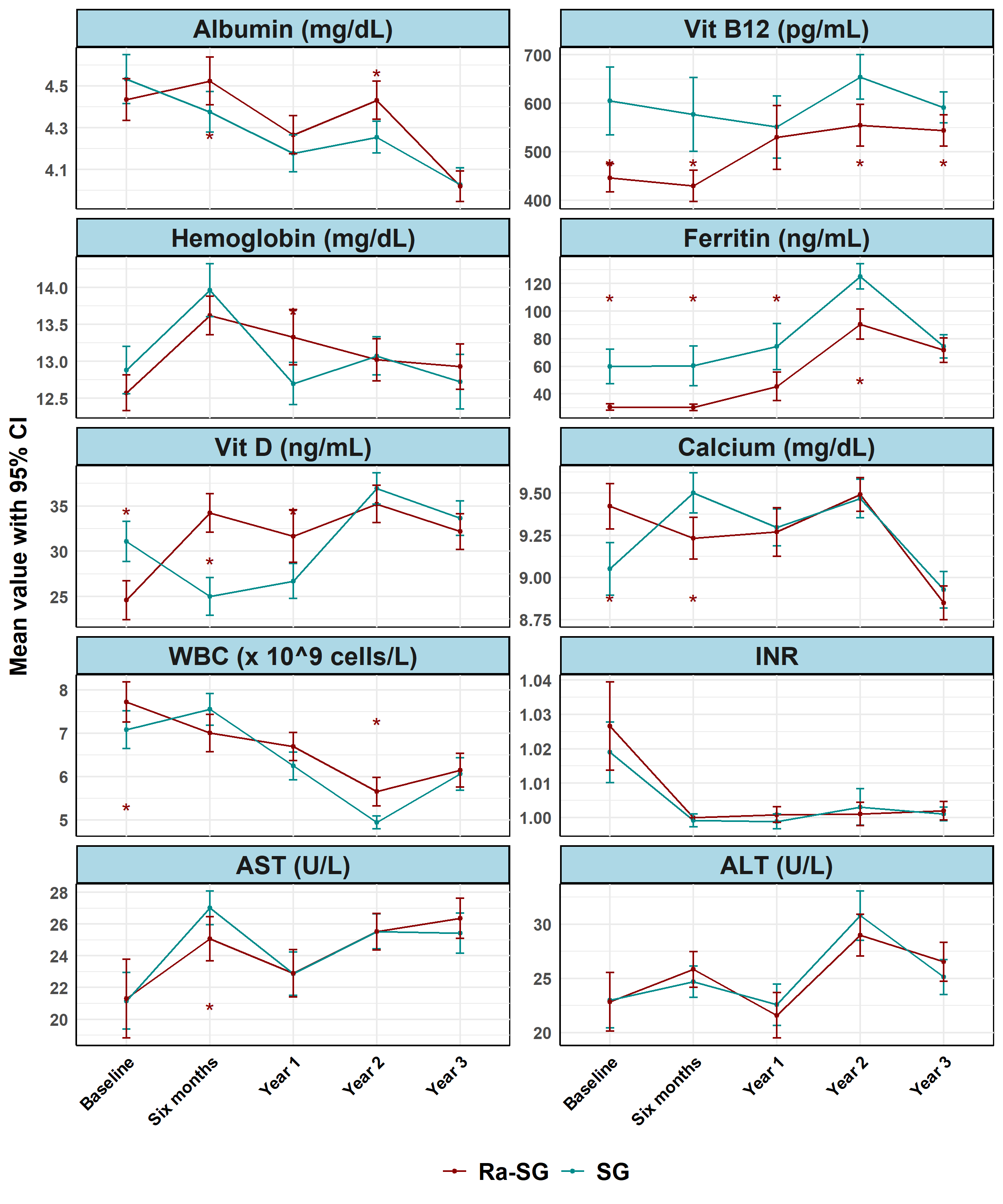


**Figure S2:** Longitudinal Changes in Selected Laboratory Parameters in SG vs. Ra-SG Groups. This figure illustrates the changes in Albumin, Vitamin B12, Hemoglobin, Ferritin, Vitamin D, Calcium, WBC, INR, ALT, and AST levels over time in patients undergoing Sleeve Gastrectomy (SG) and Ring-augmented Sleeve Gastrectomy (Ra-SG). Trends highlight significant time-specific changes and differences between the two surgical groups marked by stars to denote statistical significance.

**Figure S3**


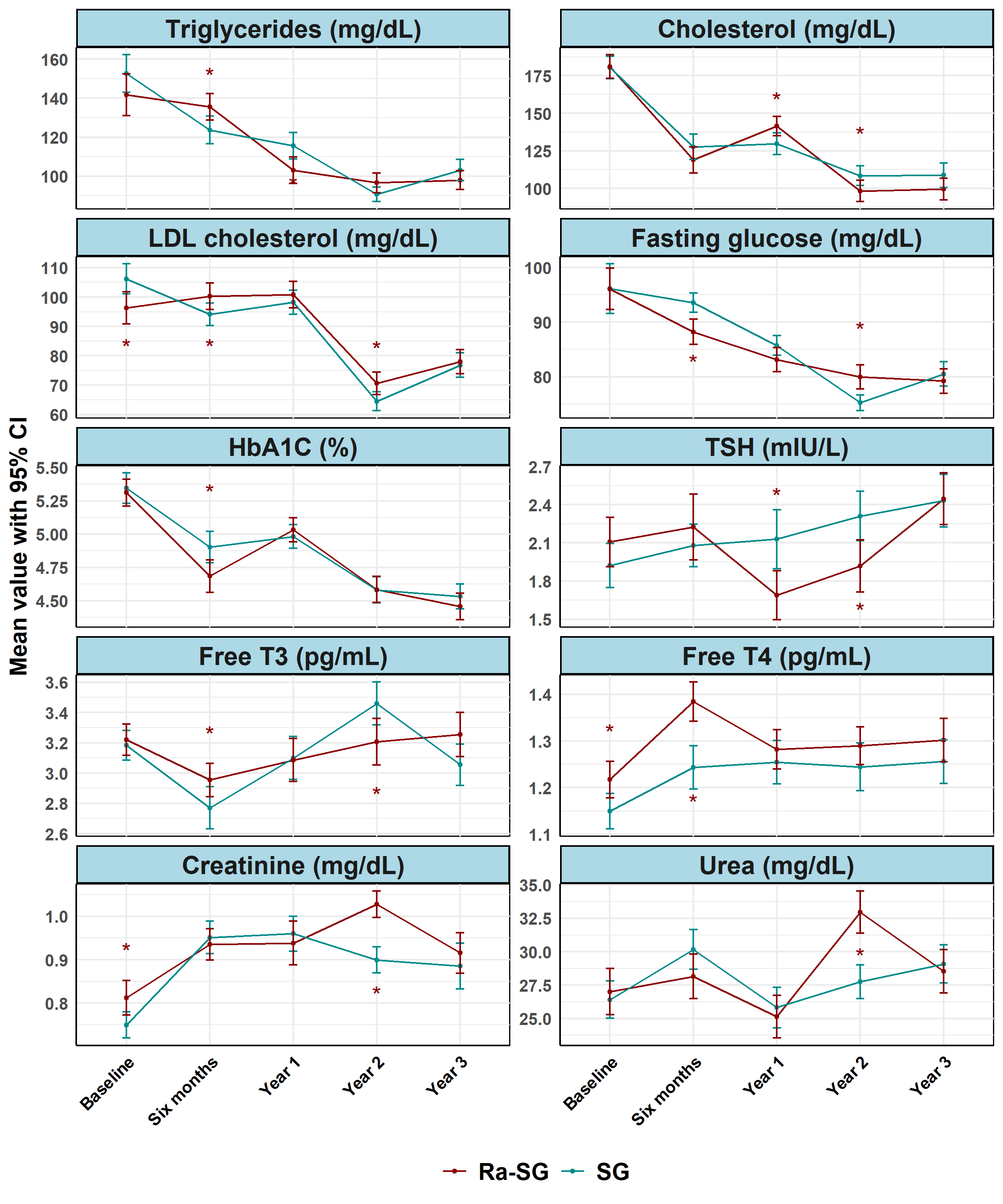


**Figure S3:** Evolution of Metabolic and Thyroid Function Parameters in SG vs. Ra-SG Groups. This figure presents the progression of metabolic and thyroid function parameters, including Triglycerides, Cholesterol, LDL cholesterol, Fasting glucose, HbA1C, TSH, Free T3, Free T4, Creatinine, and Urea, measured over time in patients who underwent Sleeve Gastrectomy (SG) and Ring-augmented Sleeve Gastrectomy (Ra-SG). Trends highlight significant time-specific changes and differences between the two surgical groups marked by stars to denote statistical significance.

**Figure S4**


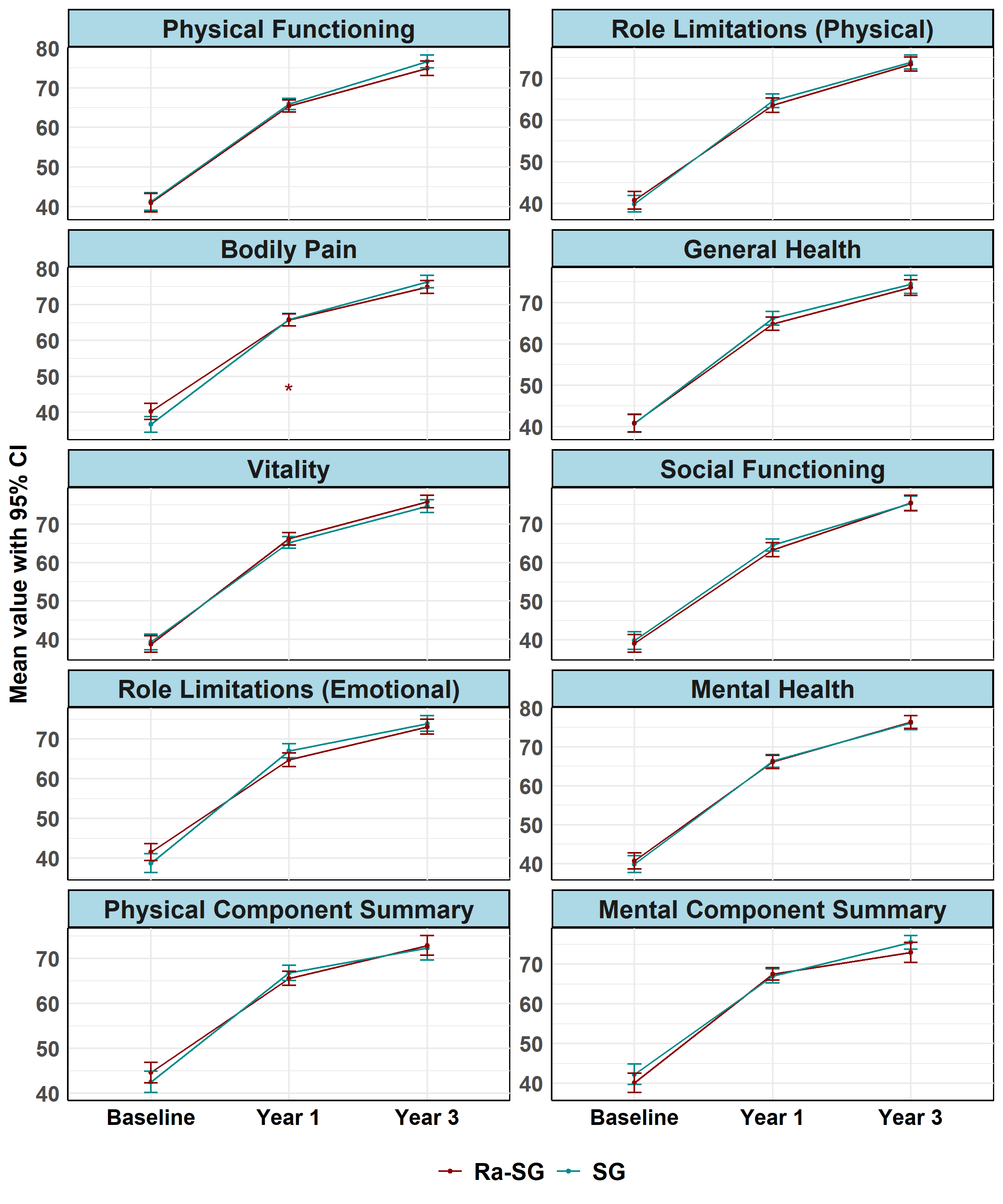


**Figure S4:** Trajectories of Health-Related Quality of Life Improvements Over Three Years Post-Surgery. This figure illustrates the progression in health-related quality of life (HRQoL) domains, as measured by the SF-36 survey, across three time points: Baseline, Year 1, and Year 3 after surgery. The graphs compare improvements in Physical Functioning, Role Limitations (Physical and Emotional), Bodily Pain, General Health, Vitality, Social Functioning, Mental Health, and the summaries for Physical and Mental Components between Sleeve Gastrectomy (SG) and Ring-augmented Sleeve Gastrectomy (Ra-SG). Mean values with 95% confidence intervals are plotted to illustrate the significant and sustained gains in HRQoL following both surgical interventions.
